# Cytokinin Signaling Downstream of the His-Asp Phosphorelay Network: Cytokinin-Regulated Genes and Their Functions

**DOI:** 10.3389/fpls.2020.604489

**Published:** 2020-11-17

**Authors:** Carlo K. Kroll, Wolfram G. Brenner

**Affiliations:** General and Applied Botany, Institute of Biology, Faculty of Life Sciences, Universität Leipzig, Leipzig, Germany

**Keywords:** cytokinin, signal transduction, downstream signaling, signaling crosstalk, feed-forward signaling, feed-back signaling

## Abstract

The plant hormone cytokinin, existing in several molecular forms, is perceived by membrane-localized histidine kinases. The signal is transduced to transcription factors of the type-B response regulator family localized in the nucleus by a multi-step histidine-aspartate phosphorelay network employing histidine phosphotransmitters as shuttle proteins across the nuclear envelope. The type-B response regulators activate a number of primary response genes, some of which trigger in turn further signaling events and the expression of secondary response genes. Most genes activated in both rounds of transcription were identified with high confidence using different transcriptomic toolkits and meta analyses of multiple individual published datasets. In this review, we attempt to summarize the existing knowledge about the primary and secondary cytokinin response genes in order to try connecting gene expression with the multitude of effects that cytokinin exerts within the plant body and throughout the lifespan of a plant.

## Introduction

The plant hormone cytokinin, regulates a wide range of processes in plants, ranging from development (growth, meristem activity, vascular development) over metabolism and physiology (source–sink relationships, secondary metabolism) to environmental interactions (both biotic and abiotic) (Mok and Mok, 2001; Argueso et al., 2009; Werner and Schmülling, 2009; Kieber and Schaller, 2014, 2018; Cortleven et al., 2019; Wybouw and De Rybel, 2019).

The immediate-early cytokinin signaling network is an extended version of the two-component signaling system known in prokaryotes. Besides having receptors (histidine kinases, HK) and transcription factors (type-B response regulators, RRB) like the original prokaryotic system, this plant-specific multi-step His-Asp phosphorelay system is augmented by mobile signaling components traveling between cytosol and nucleus (histidine phosphotransmitters, HPT), and negative feedback regulators (type-A response regulators, RRA) (Heyl et al., 2013). The RRB transcription factors are transcriptional activators, and numerous transcriptomic studies have not found genes that are consistently negatively regulated in response to cytokinin at very early time points (Brenner et al., 2012; Brenner and Schmülling, 2015), suggesting that RRBs have no repressive function.

Among the cytokinin-induced genes, there are numerous signal transduction components such as transcription factors, protein kinases, F-box proteins, etc. (Rashotte et al., 2003; Brenner et al., 2005, 2012; Bhargava et al., 2013; Brenner and Schmülling, 2015). Some of them are immediate-early response genes, the transcripts of which have started to accumulate as early as 15 min after cytokinin treatment. Others are induced at later time points, indicating that several subsequent rounds of gene expression happen after cytokinin treatment. Additionally, few genes transducing downstream branches of the cytokinin signal were found by other means.

Some of the cytokinin-regulated signal transduction genes have been functionally characterized, a few of them in great detail, and in part focusing on aspects other than cytokinin. This review aims at summarizing the accumulated knowledge about selected signaling components downstream of the phosphorelay signaling system, at finding functional interactions between them, and at presenting hypotheses resulting from mechanistic and functional considerations.

The regulation of gene expression is obviously not the only means by which signals are transduced. However, other means such as different post-translational protein modifications are not as easily detected in a comprehensive manner. Thus, this review focuses on genes transcriptionally regulated by cytokinin and mentions other means of signal transduction only if they have been shown in the context of the respective gene.

In the following paragraphs, the collected knowledge of selected cytokinin-regulated genes will be presented in order to derive ideas about their functions and their contribution to the hormonal effects of cytokinin. The selection of genes (Table 1) is largely based on their frequency of occurrence in transcriptomic investigations of the global gene expression response to the hormone in the model plant *Arabidopsis thaliana*.

**TABLE 1:** Cytokinin-regulated genes reviewed in this article.

**Gene ID**	**Gene name**	**Cited in^*a*^**	**Function (named in publications)**	**Crosstalk with major pathways**
AT1G67110	*CYP735A2*	3, 6, 8, 9, 10, 11, 13, 15, 16	Conversion of iP-type cytokinins to tZ-type cytokinins	
AT4G29740	*CKX4*	2, 3, 4, 6, 8, 9, 10, 11, 12, 13, 15, 16	Irreversible degradation of cytokinins by cleavage	
AT5G05860	*UGT76C2*	4, 6, 8, 12, 15, 16	Irreversible inactivation of cytokinins by *N*-glucosylation Drought and osmotic stress	
AT4G23750	*CRF2*	1, 3, 4, 6, 7, 8, 9, 12, 15, 16	*De novo* SAM generation in calli Lateral root elongation Root architecture in the cold	Auxin
AT2G46310	*CRF5*	3, 4, 6, 7, 8, 9, 15, 16	Development of embryos, cotyledons and leaves	Auxin
AT3G61630	*CRF6*	12, 13, 15	Leaf senescence	Auxin
AT1G68360	*GIS3*	8, 10, 12, 13, 15, 16	Trichome cell differentiation Root hair differentiation	Gibberellin
AT1G16530	*LBD3/ASL9*	3, 6, 8, 10, 11, 15, 16	Leaf development	
AT1G31320	*LBD4*	13, 15, 16	Cambium activity, vascular development, xylem differentiation	
AT2G21650	*RL2/MEE3/RSM1*	3, 6, 8, 15	Endosperm development Gravitropism Photomorphogenesis Embryo development Floral development Seed germination Response to salinity	Auxin ABA
AT4G26150	*CGA1/GNL/GATA22*	5, 6, 8, 15	Chloroplast biogenesis Chloroplast proliferation Chlorophyll biosynthesis Leaf senescence Flowering time Phyllotaxis Branching Floral development Silique length Nitrate assimilation	Light Gibberellin Auxin
AT1G13740	*AFP2*	5, 8, 13, 15	Seed dormancy Flowering time	ABA
AT3G44326	*CFB*	14, 15, 16	Sterol biosynthesis	
AT1G78580	*TPS1*	3, 8, 15	Trehalose-6-phosphate homeostasis Primary metabolism Sink–source relations	
AT2G22860	*PSK2*	3, 5	Cell division and proliferation Adventitious organ formation Pollen germination and growth Chlorophyll biosynthesis Differentiation of tracheary elements Stress-induced senescence	
AT2G17500	*PILS5*	7, 12, 13, 15, 16	Negative regulator of auxin signaling	Auxin
At2g34350		3, 15	Biotic and abiotic stress (?)	JA
At1g11670	*DTX36*	15, 16	Toxin and heavy metal efflux (?) Cell cycle (?)	Phytochrome
AT2G17820	*AHK1/HK1*	14, 15	Osmosensor	ABA
At3G51660	*MDL3*	5, 15, 16	Response to pathogens (?) Response to stress (?)	

*^*a*^ Numbers refer to the following publications: 1 Che et al., 2002; 2 Hoth et al., 2003; 3 Rashotte et al., 2003; 4 Kiba et al., 2004; 5 Brenner et al., 2005; 6 Kiba et al., 2005; 7 Rashotte et al., 2006; 8 Lee et al., 2007; 9 Taniguchi et al., 2007; 10 Yokoyama et al., 2007; 11 Argyros et al., 2008; 12 Heyl et al., 2008; 13 Brenner and Schmülling, 2012; 14 Brenner et al., 2012; 15 Bhargava et al., 2013; 16 Brenner and Schmülling, 2015.*

## Genes Involved in Cytokinin Homeostasis

### Cytokinin Biosynthesis and Activation

*CYP735A2* is a gene encoding a cytochrome p450 family protein with trans-hydroxylation enzyme activity forming *trans*-zeatin (tZ)-type cytokinins from N^6^(Δ^2^-isopentenyl) adenine (iP)-type cytokinins (Takei et al., 2004b; Kiba et al., 2013). This change of the side-chain structure is relevant for the biological activity of the respective cytokinin derivative (Schmitz et al., 1972; Mok et al., 1978) and their route of transportation via phloem or xylem (Takei et al., 2001a; Hirose et al., 2007; Kudo et al., 2010; Kiba et al., 2013). Previous studies showed that the *CYP735A2* transcript is induced by all forms of active cytokinins including the synthetic cytokinin BA, while the transcript of the paralog *CYP735A1* is insensitive to cytokinin (Takei et al., 2004b; Brenner et al., 2012; Bhargava et al., 2013). The CYP735A2 promoter harbors several core motifs and one extended motif binding type-B RRs (Brenner et al., 2012; Brenner and Schmülling, 2015), linking it with immediate-early cytokinin signaling network. Both CYP735A enzymes can be inhibited by uniconazole (Sasaki et al., 2013).

*CYP735A2* is mainly expressed in roots (Takei et al., 2001b, 2004b; Schmid et al., 2005). Higher expression levels were also found during petal differentiation, in hypocotyls and in the leaf-forming structures of the shoot apical meristem (Schmid et al., 2005). The encoded protein is predicted to be localized in mitochondria and extracellular regions. A proteomic study has found the protein in the plasmodesmata (Fernandez-Calvino et al., 2011).

*CYP735A2* is regarded as one of the major genes in maintaining the homeostasis of active cytokinins (Wang et al., 2013). This was concluded after studies with an *ugtc76c1* mutant showed attenuated N-glycosylation of tZ and iP, but stable tZ and iP content and normal developmental phenotypes. The upregulation of *CYP735A2* is the likely reason for that stable homeostasis.

*CYP735A2* is strongly upregulated by increases of the nitrate concentration in the medium (Ramireddy et al., 2014). In contrast, increased phosphate availability, acidity, and osmotic stress downregulate *CYP735A2* expression (Ramireddy et al., 2014). The CYP735A2 enzyme produces tZ-type cytokinins predominantly in the root, which are then transported to the shoot, promoting shoot growth (Takei et al., 2004b; Hirose et al., 2007; Kiba et al., 2013). Cytokinin has been proven to be one of the systemic signals of nitrogen availability in the soil (Krouk et al., 2011; Ruffel et al., 2011; Poitout et al., 2018; Vega et al., 2019). Therefore, it is concluded that *CYP735A2* may be the main regulator of that systemic signal (Ramireddy et al., 2014). However, CYP735A2-produced tZ is not the only root-to-shoot nitrate signal since cytokinin-independent signaling by mobile peptides has also been found (Ruffel et al., 2016). In addition to being a long-distance signal promoting shoot growth in the presence of nitrate, cytokinin directly influences root system architecture by suppressing root growth and branching (Ramireddy et al., 2014).

*CYP735A2* is strongly upregulated by cytokinin in roots. This can be regarded as part of a feed-forward loop. Such feed-forward loops tend to increase the signal through itself. On the other hand, cytokinin signaling involves numerous feedback loops mediated through type-A response regulators or cytokinin-degrading enzymes (e.g., *CKX4*, *UGT76C2*). Temporally separated counteracting feed-forward and feed-back loops are frequently observed in developmental biology as they help establish patterning by promoting steeper gradients of morphogens between different domains. In this scenario, a feed forward loop may help establish a state of no return, fixing the developmental fate of a cell or a group of cells. In terms of long-distance signaling, a feed-forward loop may conceptually be a signal enhancing mechanism to increase the speed of signal propagation. In this case, that concept would be realized by a process in which tZ-activated CYP735A2 successively synthesizes tZ in the tissue at the arriving tZ signal. Such a mechanism may be faster than the process relying on transport with the water stream in the xylem, which is dependent on water evaporation of the upper shoot and may therefore, under conditions of little evaporation, be quite slow.

### Cytokinin Deactivation and Degradation

*CKX4* encodes one of the seven cytokinin oxidases/dehydrogenases in Arabidopsis (Werner et al., 2001, 2003, 2006), and is the only one whose transcript levels are strongly induced by cytokinin treatment (Rashotte et al., 2003; Brenner et al., 2005). CKX enzymes degrade cytokinins irreversibly by cleaving the adenine or adenosine moiety from the respective side chain. Thus, the induction of the *CKX4* gene by cytokinin may be regarded as another negative feedback mechanism superimposed to the negative feedback by type-A response regulators at the signaling level.

*CKX4* is predominantly expressed in the root cap, but also in meristemoid cells of the leaf epidermis forming stomata (Werner et al., 2003). Other authors found *CKX4* expression in a wide variety of other tissues, including the shoot apex (Schmid et al., 2005; Obulareddy et al., 2013) and in the endo-reduplicating cells of developing trichomes and stipules (Werner et al., 2006). No exact function of the *CKX4* gene could be established by analysis of single mutants as it obviously has overlapping functions with other *CKX* genes. Overexpression of *CKX4* as well as other *CKX* genes appeared to increase tolerance to drought, heat, or salt stress. Apparently, decreased levels of iP and tZ, which are the main substrates of CKX4 (Gajdošová et al., 2011), play a major role in establishing drought, heat, or salt stress tolerance (Wang et al., 2020). It was also determined that CKX4 plays a role in the pathogen-induced reduction of cytokinin levels after inoculation with *Pseudomonas syringae* pv. Tomato DC3000 since the gene is induces by the phytotoxin coronatine delivered through the type III secretion system, thereby downregulating the plant defense system (Thilmony et al., 2006). Lastly, *CKX4* expression is down-regulated by IAA (Bilyeu et al., 2001; Wang et al., 2020), underlining the importance of this gene in auxin–cytokinin crosstalk.

Although the main expression domain of *CKX4* is in the root cap, it has most likely also a function in the shoot apical meristem: A *ckx3 ckx4* double mutant showed a significant increase of the meristem activity manifesting in a higher number of flowers and siliques (Bartrina et al., 2011). In the shoot apical meristem, cytokinin oxidases/dehydrogenases are involved in cytokinin homeostasis to maintain meristem activity at sustainable levels, and *CKX4* may be an active negative feedback regulator in this signaling circuitry due to its responsiveness to cytokinin.

The CKX4 protein is most likely secreted into the apoplast as it has corresponding sequence features and is also secreted when expressed in the yeast *P. pastoris* (Bilyeu et al., 2001; Werner et al., 2003). Computational localization predicts the protein also to localize in the ER. Other CKX proteins were predicted to be localized in the mitochondria (Schmülling et al., 2003), or found in the vacuole (Šmehilová et al., 2009; Kowalska et al., 2010), and in the cytoplasm (Zürcher and Müller, 2016). iP-ribotides and tZ-ribotides are the predominant long-range transport forms of cytokinin, but their respective locations of biosynthesis and directions of transport differ fundamentally: While tZ-type cytokinins move from the root to the shoot in the xylem, iP-type cytokinins are transported rootward through symplastic connections in the phloem (Takei et al., 2001b; Corbesier et al., 2003; Matsumoto-Kitano et al., 2008; Shimizu-Sato et al., 2008; Kudo et al., 2010; Bishopp et al., 2011). Given their different subcellular locations, it is likely that different CKX genes are specialized in degrading different types of cytokinins with differing functions.

*CKX5* is another cytokinin oxidase/dehydrogenase gene mainly expressed in the testa and in old leaves and primarily appears to have functions in germination, senescence and flowering (Gajdošová et al., 2011; Klepikova et al., 2016). Like CKX4, the CKX5 protein is localized in the ER and secreted into the apoplast (Werner et al., 2003; Zürcher and Müller, 2016). CKX5 is not very specific with regards to its substrate and metabolizes, in contrast to CKX4, *cis*-zeatin and *cis*-zeatin riboside quite efficiently (Gajdošová et al., 2011). These findings underline the assumption that different CKX enzymes are degrading different forms of cytokinin. Differential tissue-specific expression patterns suggest that the degradation of specific cytokinins happens in specific parts of the plant. Complex glycosylation patterns were found, and it has been speculated that these may be responsible for the regulation of enzymatic activity, protein stability, pH optimum, or subcellular localization (Schmülling et al., 2003; Werner et al., 2003; Gajdošová et al., 2011).

Another cytokinin-deactivating gene transcriptionally induced by cytokinin is *UGT76C2*, which encodes a cytokinin N- glycosyltransferase of *Arabidopsis thaliana* (Wang J. et al., 2011; Li et al., 2015; Šmehilová et al., 2016). It is one out of three UGTs having the ability to deactivate cytokinin *in vivo*. *UGT76C2* is an immediate-early cytokinin response gene (Kiba et al., 2004, 2005; Lee et al., 2007; Heyl et al., 2008), and its gene product was shown to be located in the cytosol (Šmehilová et al., 2016). The gene shows a spatio-temporal expression pattern in plants with high expression levels in roots, hypocotyls, cotyledons, young leaves, young lateral roots and immature seeds, but low expression levels in inflorescences and other tissues (Wang J. et al., 2011).

In comparison to the wild type, the amount of cytokinin N-glycosides is reduced in *ugt76c2* loss-of-function mutant plants and increased in plants overexpressing *UGT76C2*. The content of active cytokinins is increased in *ugt76c2* mutant plants, which is reflected by pertinent phenotypes in roots (root length and lateral root density), leaves (chlorophyll retention in detached leaves kept in the dark), and seeds (seed size) correlating with typical cytokinin functions (Wang J. et al., 2011). Being a cytokinin-deactivating gene, *UGT76C2* influences the expression of other cytokinin homeostasis and signaling genes: In *UGT76C2*-deficient plants, the positive regulators of the cytokinin status *AHK2*, *AHK3*, *ARR1*, and *IPT5* are down-regulated, while the negative regulator *CKX3* is upregulated (Wang J. et al., 2011). Generally, loss of UGT enzyme activity tends to be compensated by an increased *CKX* gene activity (Šmehilová et al., 2016). Transgenic plants overexpressing the *UGT76C2* gene show enhanced tolerance to water deficit suggesting a function of UGT76C2 in drought stress adaptation (Li et al., 2015).

In summary, *CKX4*, *CKX5*, and *UGT76C2* show crosstalk signaling with other cytokinin homeostasis and signaling genes, such as the receptor genes *AHK2*, *AHK3*, *AHK4* and the response regulator genes *ARR1* and *ARR2*, suggesting a complex network of balancing feed-forward and feed-back loops, and signal attenuation events that may be differentially shaped depending on cell type, tissue or the underlying conditions.

## Transcription Factor Genes Regulated by Cytokinin

The Arabidopsis genome harbors more than 1,600 genes encoding transcription factors, more than 5% of the protein-coding genes. Based on their phylogenetic relationship they can be grouped into at least 11 major families. Members of at least four families, ERF/AP2, zinc finger, LBD/ASL, and MYB, are directly or indirectly transcriptionally regulated by cytokinin.

### Cytokinin-Responsive *CRF* Genes Have Roles in Diverse Areas Such as Stress Response and Development

According to sequence similarity, *CRF2*, *CRF5*, and *CRF6* are the three cytokinin-responsive genes of a group of six identified as the CRF (Cytokinin Response Factor) subset of ERF/AP2 transcription factor genes (Rashotte et al., 2006; Rashotte and Goertzen, 2010; Cutcliffe et al., 2011; Jeon et al., 2016). Among other functions, they play a major role in establishing adjustments to pathogens, wounding and cold (Müller and Munné-Bosch, 2015; Sun X. et al., 2020). All three of the encoded proteins contain a highly conserved DNA-binding AP2 domain in the central region. This domain is around 60 amino acids long (Weigel, 1995; Cutcliffe et al., 2011) and directly binds to the GCC-Box, which appears to be the key motif in the promoters of ethylene-responsive genes (Hao et al., 1998; Sakuma et al., 2002; Rashotte and Goertzen, 2010; Sun X. et al., 2020). Additionally, they have a C-terminal MAPK phosphorylation site and an N-terminal CRF domain. Deletion constructs lacking the C-terminal domains of CRF5 demonstrated that the AP2 domain is required for target gene transcription (Cutcliffe et al., 2011; Striberny et al., 2017).

CRF proteins form dimers among each other, with the CRF domain functioning as the sole dimerization domain (Cutcliffe et al., 2011). They also interact with all histidine phosphotransmitter proteins and some of the type-A and type-B response regulator proteins of the TCS pathway, probably also by means of the CRF domain, but with none of the cytokinin receptors. Specific interactions between response regulators and CRF proteins were reported for CRF2 with ARR1, ARR7, ARR10, and ARR12, for CRF5 with ARR1 and ARR12, and for CRF6 with ARR6, ARR9, ARR10, and ARR11 (Rashotte et al., 2006; Cutcliffe et al., 2011; Jeon et al., 2016; Zwack et al., 2016). For *CRF2*, and *CRF5*, multiple type-B RR binding motifs were found in the 5′ region (Brenner et al., 2012; Brenner and Schmülling, 2015).

Outside of the cytokinin signaling network, CRFs influence the auxin transport machinery. Transcription of the two auxin efflux carrier genes *PIN1* and *PIN7* is directly up-regulated by CRFs binding to PIN CYTOKININ RESPONSE ELEMENTs (PCREs) in the promoter regions of *PIN1* and *PIN7*. Consequently, plants lacking CRF activity show aberrations in developmental patterning consistent with abnormal auxin distribution. Investigations of the root suggested that CRFs fine-tune root growth and development (Šimášková et al., 2015).

As demonstrated by mutant phenotypes, the CRF proteins act as developmental regulators in embryos, leaves, and cotyledons (Rashotte et al., 2006). Gene expression data suggest that *CRF2* is important for root development (Schlereth et al., 2010), highly expressed in seeds imbibed for 1 day, and moderately expressed in cotyledons and roots of 1-day-old seedlings, young leaves, seed forming organs and developing seeds (Klepikova et al., 2016). *CRF5* appears to have it highest expression rate in the shoot apex, in the female floral organs (particularly in the ovules), in mature seeds, in the root, and in the axis of the inflorescence. *CRF6* has its highest expression levels in petals, carpels, the first internode and in the mature leaves. It is not expressed in the embryo so that the first expression of *CRF6* is shown in the cotyledon of a 1-day-old seedling. Of all three cytokinin-regulated CRFs, *CRF2* has the highest expression level (Klepikova et al., 2016).

There is strong evidence that CRF2 plays a crucial role in the MONOPTEROS (MP) signaling pathway during *de novo* shoot apical meristem (SAM) generation in calli. The transcription factor MP directly binds to the *CRF2* promoter and positively regulates its expression, positioning CRF2 as a downstream signaling molecule of MP (Schlereth et al., 2010; Ckurshumova et al., 2014). Loss of function of *CRF2* totally abolished the increased shoot formation present in calli expressing a constitutively active variant of MP (Ckurshumova et al., 2014). The finding that CRF2 is a strong positive regulator of shoot regeneration from calli, more precisely the *de novo* establishment of SAMs, strongly suggests a role as a mediator of the cytokinin signal in this cytokinin-dependent process.

Besides its role in fine-tuning root growth and *de novo* SAM generation, the transcription factor CRF2 is involved in lateral root (LR) elongation. Together with CRF3, it promotes LR elongation, which is strongly reduced under cold stress in *crf2 crf3* double mutants (Jeon et al., 2016). Interestingly, cold-induced up-regulation of the *CRF2* transcript is partially dependent on the two-component signaling system (Jeon et al., 2016), indicating convergence of multiple signaling pathways upstream of the *CFR2* promoter. Whereas cytokinin inhibits LR initiation (Riefler et al., 2006; Laplaze et al., 2007; Bielach et al., 2012; Chang et al., 2015), it is involved in LR elongation, and cytokinin-responsive genes, among them CRF2, are expressed in emerging lateral roots. In summary, CRF2 is involved in shaping root system architecture in response to cold, and is probably also involved in the cellular signal transduction of other root growth responses mediated by cytokinin.

Cytokinin has a major function in delaying leaf senescence (Werner and Schmülling, 2009). Recently it was found that *CRF6* has a major role in dark-induced and stress-induced senescence and is most likely part of a fine-tuning system between both senescence pathways. Among the receptors, the main mediator of this response is *AHK3* (Kim et al., 2006). Furthermore, the CRF6 protein acts as a negative regulator in developmental leaf senescence and senescence caused by oxidative stress (Zwack and Rashotte, 2013; Zwack et al., 2016). From experiments with H_2_O_2_ it was concluded that *CRF6* has a function as a transcriptional suppressor repressing the expression of the type-A RRs *ARR6*, *ARR9*, and of the type-B RR *ARR11* in terms of signaling, *LOG7* in terms of cytokinin biosynthesis and *ABCG14* in terms of cytokinin transport (Zwack et al., 2016). The role of cytokinin and its downstream signaling components in alleviating diverse stresses is still not fully investigated. Research into this topic may lead to findings of potential importance in agriculture when applied in green biotechnology (Gan and Amasino, 1995).

In summary, cytokinin-regulated CRFs appear to mediate a number of cytokinin-related plant responses in different organs. As most of these CRFs are also regulators in other signaling pathways, they can be regarded as hubs for crosstalk and signal integration between cytokinin and stress-related signaling.

### *GIS3*: A Link Between Cytokinin and Trichome/Root Hair Development

The protein encoded by *GIS3* (AT1G68360, *GLABROUS INFLORESCENCE STEMS*) is a member of the C2H2-type Zinc finger family of transcription factors and is induced by cytokinin as early as 15 min (Bhargava et al., 2013; Brenner and Schmülling, 2015). It is a direct target of the type-B response regulator ARR10 (Zubo et al., 2017). The C2H2 subfamily of zinc finger transcription factors contains the GIS subfamily made up of *ZFP5*, *ZFP6*, *ZFP8*, *GIS*, *GIS2*, and *GIS3* (Sun et al., 2015), two of which (*ZFP6*, *ZFP8*) were also reported as direct targets of ARR10 (Zubo et al., 2017). The main function of *GIS3* is in trichome development where it is a positive regulator of trichome cell differentiation and morphogenesis (Sun et al., 2015; Han et al., 2020). *GIS3* is primarily expressed in these tissues but additionally in roots (Schmid et al., 2005; Sun et al., 2015).

Cytokinin is a positive regulator of trichome formation in Arabidopsis, and it is even able to induce trichome formation when applied to organs that normally do not form trichomes, such as floral organs (Greenboim-Wainberg et al., 2005). Cytokinin signaling promoting trichome differentiation is transduced through two other C2H2 zinc finger proteins, ZFP8 and GIS2, the latter being a cytokinin-inducible gene itself (Gan et al., 2007b). GIS3 acts upstream of GIS, GIS2 and ZFP8 to induce trichome development by binding to their promoters (Gan et al., 2007a,b; Sun et al., 2015). It was shown that the Gibberellin-activated signaling pathway plays a key role for trichome development (Gan et al., 2007b; Sun et al., 2015). Thus, GIS3 appears to be the signaling component that feeds the cytokinin signal into the module consisting of GIS, ZFP8, and GIS2 to integrate cytokinin and gibberellin signaling.

Another function of certain C2H2 zinc finger transcription factors regarding the development of epidermal layers is in root hair development in Arabidopsis, integrating cytokinin and gibberellin signals (Han et al., 2020). Consequently, the gene is mainly expressed in root hair cells (Zhou Z.-Y. et al., 2011; An et al., 2012) but additionally it is involved in initiation of inflorescence trichomes in response to gibberellin (Zhou Z.-Y. et al., 2011). Similar to trichome development, *GIS3* functions upstream of GIS, GIS2 and ZFP8, the latter being again directly targeted by GIS3 (Zhou Z.-Y. et al., 2011).

The involvement of virtually identical signaling molecules in the development of (unicellular) trichomes and root hairs in Arabidopsis underlines the idea that both structures are developmentally related. Consequently, cytokinin plays the same promoting role in the formation of both trichomes and root hairs.

### Cytokinin-Regulated Genes Encoding LOB Domain Proteins Involved in Secondary Growth and Vascular Development

*LATERAL ORGAN BOUNDARY DOMAIN* (*LBD*) genes encode a plant-specific transcription factor family whose first discovered member *LOB* shows a ring-shaped expression pattern around the sites where lateral organs emerge from an axis (Shuai et al., 2002). These genes are also referred to as *ASL* (*AS2-like*) genes, based on their sequence similarity to ASYMMETRIC LEAVES2 (Iwakawa et al., 2002).

The *LBD3*/*ASL9* transcript was found to be induced by cytokinin in a number of transcriptomic studies (Rashotte et al., 2003; Kiba et al., 2005; Bhargava et al., 2013), but by no other hormone (Naito et al., 2007). Consistent with that, its promoter contains type-B response regulator binding sites (Brenner and Schmülling, 2015). No other LBD gene was rapidly responsive to cytokinin (Naito et al., 2007).

Another LBD gene regulated by cytokinin, albeit at later stages of the response (2 h after induction and later), is *LBD4* (AT1G31320). Unlike most cytokinin-responsive genes, which are regulated in the same way in root and shoot, *LBD4* is specifically upregulated in the root but not in the shoot (Brenner and Schmülling, 2012). *LBD4* was identified as part of a feed-forward loop in a transcriptional network analysis to identify signaling mechanisms controlling vascular development (Smit et al., 2020). This network transduces the signal of TDIF, a mobile CLE peptide perceived by the PXY receptor, leading to the upregulation of several WOX genes (Hirakawa et al., 2008, 2010; Etchells et al., 2013; Morita et al., 2016; Zhang et al., 2016). The ligand-receptor pair of TDIF and PXY is part of a regulatory loop between phloem and cambium controlling xylem differentiation through another transcription factor, BES1 (Kondo et al., 2014).

Both *LBD3* and *LBD4*, the two closest relatives among the LBD genes, are redundantly involved in vascular development as shown by mutant and overexpression analysis (Smit et al., 2020). Remarkably, *LBD4* is expressed at the phloem-procambium boundary, in accordance with the general expression pattern of genes of the LBD family at organ boundaries. It is hypothesized that LBD4 may function as a boundary regulator or as an enhancer of cell divisions at the phloem side of the procambium, or to have both functions. Since redundancy of *LBD3* and *LBD4* was determined, it is very likely that LBD3 acts in the same way. Both genes may act as a signaling hub feeding the cytokinin signal into the system controlling vascular development and cambial activity with *LBD4* acting as a factor differentiating between root and shoot.

### *MEE3*: Signaling Hub Coupling Cytokinin With Auxin and ABA Signal Transduction

*MEE3* (AT2G21650, *MATERNAL EFFECT EMBRYO ARREST 3*), also known as *RSM1* or *ATRL2*, belongs to the family of MYB-related transcription factors. It is a gene responding at a later time point to a cytokinin pulse, probably not being directly coupled to the phosphorelay network by the type-B response regulators.

*MEE3* is essential for endosperm development, gravitropism, and photomorphogenesis. Additionally, it may have roles in embryo development, plant hormone interaction, floral development, and response to stress, and modulates seed germination and seedling development in response to abscisic acid and salinity (Riechmann and Ratcliffe, 2000; Pagnussat et al., 2005; Baxter et al., 2007; Hamaguchi et al., 2008; Yang et al., 2018).

During early photomorphogenesis, *MEE3* may be implicated in HOOKLESS1 (HLS1)-mediated auxin signaling, negatively regulating this pathway as a feedback regulator by a mechanism that is so far unknown (Hamaguchi et al., 2008). This observation may reflect part of the antagonistic effect of cytokinin on auxin action. The mutually inhibitory influence of the two hormones on each other’s action is known since a long time from phenotypical observations and is realized on the molecular level through hormone homeostasis and signal inhibition (Dello Ioio et al., 2008; Müller and Sheen, 2008; Schaller et al., 2015). Thus, *MEE3* may be another piece to be added to the multi-faceted auxin–cytokinin interaction network.

MEE3 binds to the *ABI5* promoter driving the expression of a transcription factor negatively regulating seed germination, major mediator of abscisic acid (ABA) signal transduction and abiotic stress response (Finkelstein, 1994; Finkelstein and Lynch, 2000; Lopez-Molina et al., 2001; Nakamura et al., 2001; Yang et al., 2018). In addition, MEE3 physically interacts with the transcription factor HY5, which promotes photomorphogenesis and activates *ABI5* expression (Alabadí and Blázquez, 2008; Yang et al., 2018). This interaction between MEE3, HY5, and the *ABI5* promoter appears to modulate the sensitivity of several abscisic acid (ABA)-dependent processes to the hormone. This way, cytokinin signaling couples into the ABA and abiotic stress response pathway by means of regulating *MEE3* expression.

All of the above leads to the conclusion that regulation of ABA-, auxin- and abiotic stress response may be partially mediated by *MEE3* as a secondary response gene of cytokinin during early morphogenesis (Hamaguchi et al., 2008; Yang et al., 2018).

### LLM Domain-Containing GATA Transcription Factors Mediate Multiple Developmental Processes and Promote Chloroplast Development

GATA transcription factors belong to one of four subfamilies of the C2C2 zinc finger proteins. Characteristically, they bind to the consensus sequence (T/A)GATA(G/A), which was found in the promoters of many light-regulated genes (Teakle et al., 2002; Reyes et al., 2004). Among them, *CGA1*/*GNL*/*GATA22* was found to be transcriptionally regulated by cytokinin in a number of transcriptomic studies (Kiba et al., 2004; Brenner et al., 2005; Bhargava et al., 2013) as an early-responding gene, probably directly activated by type-B response regulators. In addition to cytokinin, the *CGA1* transcript is also regulated by nitrate (Price et al., 2004; Scheible et al., 2004; Wang et al., 2004; Bi et al., 2005), (red) light (Manfield et al., 2007), and sugar (Wang et al., 2003; Price et al., 2004; Scheible et al., 2004), and is under the control of the circadian clock (Harmer et al., 2000; Alabadì et al., 2002; Manfield et al., 2007). Although *CGA1* is co-regulated with seven other GATA transcription factor genes (*GATA15*, *GATA16*, *GATA17*, *GATA17L*, *GATA21*/*GNC*), all of which contain an LLM (Leu-Leu-Met) domain (Ranftl et al., 2016), it is special with regards to its particularly strong reaction to cytokinin. Of these, the two paralogs *GATA21*/*GNC* and *GATA22*/*GNL*/*CGA1* are repressed by the homeotic floral organ identity transcription factors AP3 and PI (Mara and Irish, 2008). Higher-order mutants of these transcription factor genes showed defects in several cytokinin-regulated developmental processes such as phyllotaxis, cytokinin-induction of leaf greening and suppression of chlorophyll degradation during leaf senescence, branching and plant height, the number of floral organs and silique length (Ranftl et al., 2016).

*CGA1* has multiple roles in plant development and physiology. In terms of crosstalk with other hormones, it represses gibberellin signaling downstream of the DELLA proteins and PIFs (Richter et al., 2010), enabling a negative regulation of gibberellin signaling by cytokinin. Consistently, plants overexpressing CGA1 show an altered timing of numerous developmental events such as germination, leaf production, flowering and senescence (Hudson et al., 2011). CGA1 was suspected as a point of convergence of cytokinin, light, and gibberellin signaling (Köllmer et al., 2011). The repressive effect of *CGA1* on flowering time is mediated by direct transcriptional repression of the flowering time regulator *SOC1*, simultaneously influencing greening (Bastakis et al., 2018) and cold tolerance (Richter et al., 2013a). In addition, auxin signaling converges at *CGA1*, repressing its expression through ARF7 (Richter et al., 2013b).

Mutant analysis revealed that CGA1 promotes chlorophyll biosynthesis by modulating the expression of a number of chlorophyll biosynthesis genes (Mara and Irish, 2008; Hudson et al., 2011). However, not only chlorophyll biosynthesis is regulated by *CGA1*, but chloroplast proliferation in all aspects, development, growth, and division. For these processes, *CGA1* was assigned the role of a master regulator because overexpression causes ectopic chloroplast development even in roots or in darkness (Chiang et al., 2012; Zubo et al., 2018). From the analysis of mutants, it was also concluded that the positive effect of cytokinin on chloroplasts is at least partially transduced through CGA1. During wound-induced root greening, *CGA1* and *GNC* are important factors transducing the cytokinin signal, but the exact way how CGA1 and other GATA transcription factors induce the transcription of photosynthesis-related genes is not known (Kobayashi and Iwase, 2017; Kobayashi et al., 2017). These findings are consistent with the observation that cytokinin shifts the root transcriptome toward a more shoot-like profile, which may be largely due to chloroplast genes becoming expressed in the root after an extended period of cytokinin treatment (Brenner and Schmülling, 2012).

In terms of metabolism, CGA1 positively regulates the expression of *GLU1* encoding the chloroplast-localized GLUTAMATE SYNTHASE1, the primary enzyme controlling nitrogen assimilation in green tissue and providing substrate for chlorophyll biosynthesis (Hudson et al., 2011). This may be another section of nitrate signaling mediated by cytokinin, coupling into processes related to greening and photosynthesis.

## Signaling by Targeted Protein Degradation

### *AFP2*: An ABA Signaling Component Targeting ABI5 for Proteasomal Degradation

*AFP2* (AT1G13740, *ABI FIVE-BINDING PROTEIN*) belongs to a small family of five genes in Arabidopsis (Garcia et al., 2008), whose members bind to the transcription factor and key regulator of the ABA response ABI5, thereby attenuating the ABA response by targeting ABI5 for ubiquitin-mediated degradation (Lopez-Molina et al., 2003). All these proteins share three conserved domains of unknown function (Garcia et al., 2008). Additionally, the transcriptional repression of ABI5 target genes may be mediated by recruitment of a co-repressor of the TOPLESS family (Pauwels et al., 2010; Causier et al., 2012; Lynch et al., 2017). AFP proteins also interact with themselves and other members of the AFP family, and, remarkably, also with histone deacetylases, providing another level of gene regulation by chromatin modification. AFP2 has also emerged as a regulator for breaking heat-induced secondary seed dormancy (Chang et al., 2018) and as a factor delaying flowering time (Chang et al., 2019).

Cytokinin negatively regulates ABA-dependent responses such as drought and salt tolerance (Tran et al., 2007). Thus, cytokinin-induced upregulation of AFP2 may be one of the molecular links mediating the negative influence of cytokinin on ABA signaling. Another mechanism of cytokinin-ABA signaling crosstalk is the direct interaction of several type-A RRs with ABI5, inhibiting its function as a transcription factor (Wang Y. et al., 2011).

### *CFB*: A Cytokinin-Regulated Gene Directly Interfering With a Key Enzyme of Sterol Biosynthesis

*CFB* (At3G44326, *CYTOKININ-REGULATED F-BOX PROTEIN*) has emerged as one of the most robustly upregulated genes after cytokinin treatment in meta analyses of microarray experiments and RNA-Seq transcriptomics (Bhargava et al., 2013; Brenner and Schmülling, 2015). It is an early-responding gene and as such probably directly activated by type-B response regulators. It encodes an F-box protein belonging to a small group of three related proteins in Arabidopsis (Brenner et al., 2017). Orthologs exist in all land plants. The group of CFB-like proteins is characterized by an F-box carrying the unique motif ILTRLDG not found in the F-box domain of any other F-box protein. In addition, the proteins possess five domains of unknown function, two highly conserved sequence motifs, and a C-terminal transmembrane domain.

The CFB protein interacts with the only cycloartenol synthase enzyme in Arabidopsis, CAS1, thereby downregulating a bottleneck step in plant sterol biosynthesis. The resulting accumulation of 2,3-oxidosqualene in young shoot tissue causes a disturbed and delayed development of chloroplasts resulting in white shoot tips. In which tissues and for what purpose a possible downregulation of sterol biosynthesis by cytokinin may be relevant for plant development or other processes is not known.

## Small Downstream Effectors

### *TPS1* and Trehalose-6-Phosphate: Cytokinin Influencing Primary Metabolism

Trehalose-6-phosphate (T6P) is a major signaling molecule in plants regulating sucrose levels, hence it is referred to as “the plant insulin.” Levels of free sucrose in tissues are regulated by the formation or degradation of starch. This regulation is governed by T6P, the levels of which are highly positively correlated to sucrose levels, leading to the formation of a homeostatic feedback regulatory circuit referred to as the sucrose-T6P nexus (Figueroa and Lunn, 2016).

T6P homeostasis is governed by two enzymatic activities, trehalose-6-phosphate synthase (TPS) for synthesis, and trehalose-6-phosphate phosphatase (TPP) for degradation. Transcriptomic experiments have revealed that genes encoding these two types of enzymes are regulated in a reciprocal manner by cytokinin: Upon cytokinin treatment, *TPS* transcripts are more abundant and *TPP* transcripts are less abundant, while in cytokinin-deficient plants the opposite is true (Brenner et al., 2005). Thus, T6P levels are likely to be increased under cytokinin treatment while T6P levels are probably reduced in cytokinin-deficient plants. T6P directs primary metabolism toward a more consumptive mode, thus a cytokinin-induced increase would be consistent with the generally proliferative action of the hormone. However, it is not clear whether *TPS1* (AT1G78580), which appears to encode the major T6P biosynthetic enzyme (Fichtner et al., 2020), and the other enzymes involved in T6P homeostasis are directly regulated by the cytokinin-dependent TCS signaling network or whether the homeostatic regulation mentioned above is an indirect effect of altered sucrose levels due to cytokinin modulating carbohydrate consumption by, e.g., growth processes. Motifs that are demonstrated to bind type-B response regulators (Franco-Zorrilla et al., 2014) or that are enriched in cytokinin-responsive promoters (Brenner and Schmülling, 2015) are present in the promoter region of TPS1, favoring the idea of direct manipulation of T6P homeostasis and the associated changes in primary metabolism by cytokinin.

### *PSK2*: Phytosulfokine as a Downstream Signal of Cytokinin Leading to Its Proliferating and Chloroplast-Promoting Action?

Phytosulfokine (PSK) is a 5 aa-long peptide sulfated at two tyrosine residues that was first identified in conditioned medium of plant cell cultures, where it is the primary signal molecule for cell-cell communication promoting callus growth. Due to that property, PSK can be regarded as a plant growth factor. There are at least five PSK precursor genes in Arabidopsis, of which the *PSK2* gene (AT2G22860) is induced by cytokinin (Rashotte et al., 2003; Brenner et al., 2005). Genes encoding proteases and tyrosylprotein sulfotransferases processing the PSK precursor proteins were also identified in the Arabidopsis genome, as well as respective receptors (Matsubayashi et al., 2006a,b).

Besides its effects on callus proliferation, PSK is also associated with a number of events associated with growth and proliferation in whole plants. The PSK transcripts in rice are highly expressed in the proliferating zones of the root and shoot meristems (Yang H. et al., 1999). PSK promotes adventitious bud formation in *Antirrhinum* (Yang G. et al., 1999), adventitious root formation from hypocotyls in cucumber (Yamakawa et al., 1998b), somatic embryogenesis (Kobayashi et al., 1999; Hanai et al., 2000; Igasaki et al., 2003), and pollen germination and growth (Chen et al., 2000; Stührwohldt et al., 2015). It also enhances chlorophyll biosynthesis in the dark such as during the night or under etiolating conditions (Yamakawa et al., 1998a; 1999). Finally, PSK promotes the differentiation of tracheary elements (Matsubayashi et al., 1999) and retards stress-induced senescence (Yamakawa et al., 1999).

Many of these PSK functions overlap with the effects observed by cytokinin and are in accordance with the generally proliferative, growth-promoting and anti-senescence action of the hormone. Thus, it is tempting to speculate that PSK may be an important downstream signal of cytokinin. The *PSK2* promoter contains several motifs either found to be bound by type-B response regulators or enriched in the promoters of other cytokinin-induced genes, encouraging investigations into the *PSK2* gene as part of the downstream signaling network of cytokinin.

## Transport Across Membranes

### *PILS5*, a Player in Cytokinin–Auxin Interactions

*PILS5* (AT2G17500, *PIN-LIKES 5*) encodes a PIN transporter-like auxin efflux carrier protein and is induced by cytokinin during the late response (≥120 min) (Brenner et al., 2005; Brenner and Schmülling, 2015). There are seven members in the PIN-LIKES family. PILS proteins have predicted topological similarities to PIN-FORMED proteins, despite the circumstance that they only share 10–18% of their sequence (Feraru et al., 2012; Sun L. et al., 2020). PILS family members were identified by the presence of the auxin carrier domain spanning nearly the whole length of the PILS protein. Due to that domain, PILS proteins are predicted to have auxin transport function (Barbez et al., 2012). However, it is difficult to pinpoint functional residues within the domain. Moreover, nothing is known about possible post-translational modifications, but generic phosphorylation sites, kinase specific phosphorylation sites and isoform variations were predicted (Blom et al., 1999, 2004). Furthermore, different numbers of serine, threonine and tyrosine phosphorylation sites were used to assign three different classes of PILS proteins. *PILS5* was grouped into class one because it has less than 10 phosphorylation sites (Feraru et al., 2012).

Interestingly and in contrast to the proper PIN transporters, the subcellular localization of PILS proteins is in the ER (Barbez et al., 2012). For that reason, expression of PILS transporters results in a retention of auxin within cells. They sequester auxin at the ER, limiting active auxin availability in the nucleus, thereby attenuating auxin signaling and decreasing cellular sensitivity to auxin (Barbez et al., 2012; Feraru et al., 2012, 2019; Béziat et al., 2017). Furthermore, it affects auxin homeostasis and signaling by regulating the auxin conjugation rate and its intracellular accumulation. Consequently, *PILS5* gain-of-function results in multiple phenotypic changes consistent with a low auxin status regarding root organ growth (lateral root formation positively, root-hair elongation negatively), growth regulation in general, as well as seedling growth and development (Barbez et al., 2012; Dal Bosco et al., 2012; Feraru et al., 2012; Barbez and Kleine-Vehn, 2013; Sun L. et al., 2020).

Phylogenetic analyses revealed that PILS proteins are probably older than PIN-FORMED proteins, hence intracellular auxin accumulation is evolutionary older PIN dependent auxin transport (Feraru et al., 2012). Nearly all family members except for PILS4 originate from lineage specific duplications. They are grouped into three different clades with PILS5 grouped into Clade III (Feraru et al., 2012).

Transcription of *PILS5* is strongly dependent on auxin, cytokinin and brassinosteroid levels (Sun L. et al., 2020). Additionally, abiotic factors such as light and temperature, repress *PILS5* expression, leading to growth effects reminiscent of a higher auxin status (Feraru et al., 2012; Béziat et al., 2017; Sun L. et al., 2020). The gene is expressed during all developmental stages, specifically in mature pollen (Klepikova et al., 2016), seedling, cauline leaves, and flowers (Barbez et al., 2012). Through the well-known antagonistic action between auxin and cytokinin, *PILS5* indirectly affects homeostasis and signaling of cytokinin (Kuderová et al., 2008; Naseem and Dandekar, 2012). That antagonism may be accomplished by auxin mediated shifts in pH that regulate cytokinin receptor activity (Werner and Schmülling, 2009). A more direct signaling mechanism is the upregulation of certain type-A response regulator genes by the auxin signal transduction (Müller and Sheen, 2008). AUXIN RESPONSE FACTOR3 represses cytokinin biosynthesis and signaling at multiple levels (Zhang et al., 2018). During plant development these interactions are important, e.g., for cell specification, growth and size of plant structures both below-ground and above-ground (Müller and Sheen, 2007; Taniguchi et al., 2007; Dello Ioio et al., 2008).

In summary, *PILS5* promotes auxin accumulation at the ER, thereby repressing auxin signaling (Barbez et al., 2012; Feraru et al., 2012; Sun L. et al., 2020). As cytokinin supposedly increases PILS5 activity by transcriptionally activating the corresponding gene, PILS5 may be one of the players that mediate the negative influence of cytokinin on auxin signaling, making it a factor in mediating crosstalk of cytokinin and auxin.

### *DTX36*: A Transmembrane Export Protein Probably Involved in Abiotic Stress Response

*DTX36* (At1g11670, *DETOXIFICATION 36*) encodes a MATE-related efflux protein located in membranes, particularly in the plasma membrane (Li et al., 2002; Gaudet et al., 2011). It is part of a gene family of at least 56 members mediating the efflux of endo- and exogenous toxic compounds and heavy metals (Li et al., 2002). Upregulated at 15 min after cytokinin treatment, *DTX36* is an early cytokinin response gene (Bhargava et al., 2013; Brenner and Schmülling, 2015), probably directly activated by type-B response regulators. Furthermore, the gene is regulated by the cell cycle peaking in the G1 phase (Menges et al., 2002). Another process during which *DTX36* is regulated is photomorphogenesis induced by the phytochrome pathway: *fhy3* and *far1* mutants show reduced *DTX36* expression (Hudson et al., 2003).

*DTX36* is expressed in nearly every structure from seed, root, shoot, leaves, to inflorescence structures (Schmid et al., 2005; Obulareddy et al., 2013). The highest expression levels were found in seeds after 3 days of soaking and in the root apex of seedlings, whereas the lowest expression levels were found in dry seeds (Klepikova et al., 2016). Generally, the expression in the roots was higher than in aboveground organs.

Cytokinin has been implicated in stress responses in numerous studies (Naseem et al., 2014; Bielach et al., 2017; Yang and Li, 2017; Huang et al., 2018; Kieber and Schaller, 2018; Cortleven et al., 2019). Here, an unspecific stress response gene is induced by cytokinin in an immediate-early fashion, further corroborating the function of cytokinin as a hormone involved in stress response.

### At2g34350: A Nodulin-Like Major Facilitator Superfamily Gene With Links to Biotic and Abiotic Stress

According to sequence similarity, the gene At2g34350 is a Nodulin-like major facilitator superfamily protein. As a member of this family, it is probably involved in transmembrane transport of hydrophilic molecules or water itself. Genes of this family are mainly associated with the response to abiotic stress, but also to biotic stress (Bezerra-Neto et al., 2019).

The gene is primarily expressed in the root apex (Klepikova et al., 2016) and is induced by cytokinin as a late (120 min) response gene (Rashotte et al., 2003; Bhargava et al., 2013). Its cytokinin-dependent expression pattern was further confirmed in plants overexpressing *ARR22*, a negative regulator of cytokinin signaling (Wallmeroth et al., 2017, 2019), where its transcript levels were lower (Kiba et al., 2004).

Furthermore, the gene is also regulated by salt stress (Sottosanto et al., 2004) and the jasmonate signaling pathway (Chini et al., 2007), corroborating the idea that it has a role in biotic and abiotic stress response. Its exact function, however, has not yet been investigated.

## Genes With Other Functions

### *AHK1*: A Probable Osmosensor

Another gene induced by cytokinin is *AHK1* (AT2G17820, *ARABIDOPSIS HISTIDINE KINASE 1*) (Brenner and Schmülling, 2012; Bhargava et al., 2013). The gene encodes a member of the histidine kinase family and is involved in response to osmotic stress, response to water deprivation, seed maturation and stomatal complex patterning (Tran et al., 2007; Wohlbach et al., 2008; Kumar et al., 2013). Unlike the three cytokinin receptors AHK2, AHK3, and AHK4, which belong to the same family, AHK1 is an osmosensor, but – lacking the cytokinin-binding CHASE domain – not a cytokinin sensor. Just like the cytokinin receptors, AHK1 acts according to the principle of histidine phosphotransfer (Urao et al., 1999).

The gene is expressed in nearly every plant structure in relatively even levels (Schmid et al., 2005; Obulareddy et al., 2013; Klepikova et al., 2016). The subcellular localization of the Arabidopsis protein and at least one of the poplar orthologs is in the plasma membrane (Caesar et al., 2011; Héricourt et al., 2013, 2016).

AHK1 is suggested to be a positive regulator in stress response through ABA-dependent and ABA-independent signaling pathways. Furthermore, it has a major role in plant growth (Tran et al., 2007). Additionally, it is a necessary player to prevent desiccation during seed development and also in vegetative tissues (Wohlbach et al., 2008). How water limitation is actually sensed is not finally clarified, however, the predicted extracellular domain is essential for its activity (Urao et al., 1999). AHK1 most likely integrates mechanisms such as sensing of cell volume, shape, turgor pressure, or macromolecular crowding to a downstream signal that is so far unknown (Kumar et al., 2013). Interestingly, it was shown in poplar that the cytokinin receptors are also able to interact with those histidine phosphotransmitter proteins through which the poplar orthologs of AHK1 signal (Héricourt et al., 2019). Conversely, the poplar orthologs of AHK1 are unable to interact with a subset of histidine phosphotransfer proteins that are uniquely interacting with the cytokinin receptors. Thus, in poplar, crosstalk can happen from cytokinin into the osmosensing pathway, but not vice versa. Similar investigations in Arabidopsis are missing.

### *MDL3*: A Gene of Unknown Function With Links to Diverse Abiotic Stresses

The *MDL3* gene (At3G51660, MACROPHAGE MIGRATION INHIBITORY FACTOR/D-DOPACHROME TAUTOMERASE-LIKE PROTEIN 3) is expressed in nearly every Arabidopsis plant structure including even plant sperm cells and guard cells (Schmid et al., 2005; Obulareddy et al., 2013; Klepikova et al., 2016). The highest expression levels were found in the petioles of senescent leaves and in the pods of older siliques (Klepikova et al., 2016). The encoded protein is an LS1-like protein belonging to the tautomerase/MIF superfamily and is localized in the peroxisomes (Reumann et al., 2007).

Proteins of this family are found in mammalian and non-mammalian organisms and are known as upstream mediators of various immune responses. In plants it most likely integrates intracellular effects and induces precursor proteins which are part of the secondary plant metabolite signaling pathway (Panstruga et al., 2015; Sparkes et al., 2017). The transcript is induced as early as 15 min after cytokinin treatment (Brenner et al., 2005; Brenner and Schmülling, 2015), by cold stress (Mori et al., 2018), osmotic stress, wounding, and UV-B radiation (Panstruga et al., 2015). The protein is most likely a part of self-protection of plants in response to pathogens and environmental stress (Reumann et al., 2007; Ascencio-Ibáñez et al., 2008; Panstruga et al., 2015; Mori et al., 2018) and therefore possibly also part of cytokinin-mediated stress responses. Its subcellular localization in the peroxisome substantiates a possible function in defense and/or detoxification mechanisms (Reumann et al., 2007).

## Discussion

In the previous paragraphs, accumulated knowledge about a selection of cytokinin-regulated genes was collected and summarized (Table 1). The selection of genes was based on the number of occurrences primary literature about cytokinin-related transcriptomic studies (Brenner et al., 2012; Bhargava et al., 2013; Brenner and Schmülling, 2015). These genes can be regarded as a subset of the most reliably cytokinin-regulated genes. We focused our selection on signaling genes, but included genes with other functions as well if significant knowledge was found in the literature.

**FIGURE 1:**
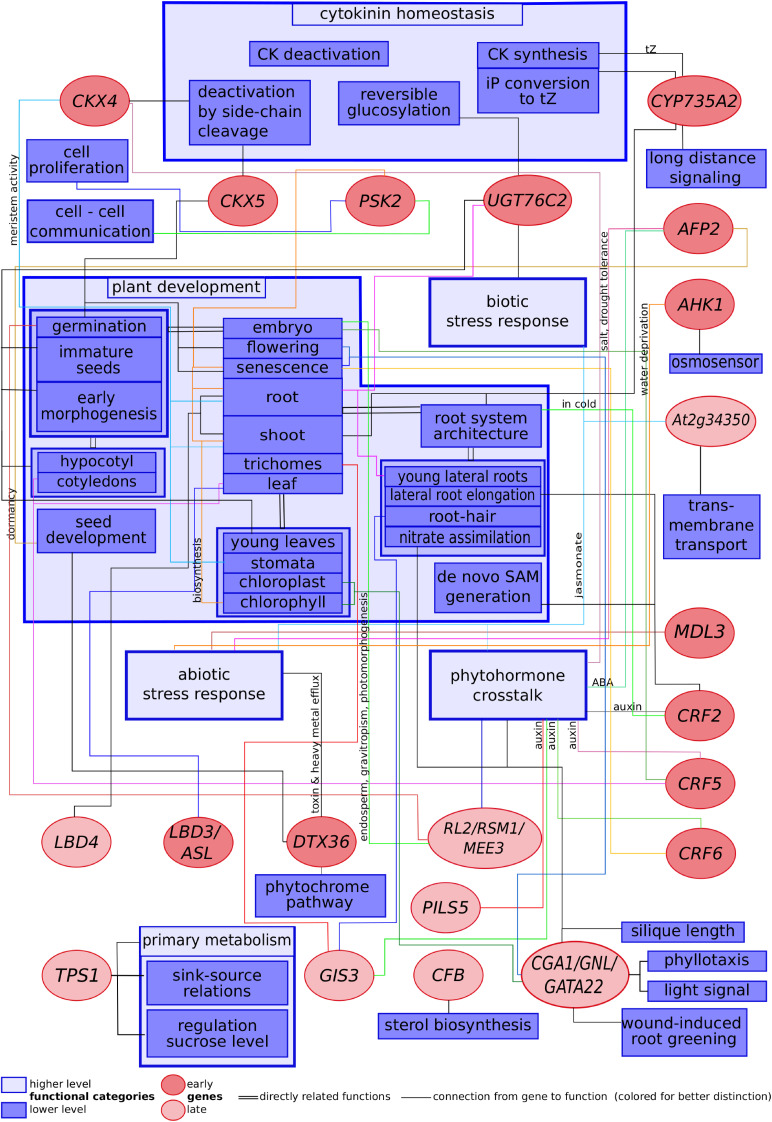
Scheme showing selected cytokinin-regulated genes related to their functions described in published literature.

Collecting information available in the literature has revealed numerous functional connections between cytokinin and processes such as plant development, primary metabolism, biotic and abiotic stress response, cytokinin homeostasis and phytohormone crosstalk. These connections between cytokinin-regulated genes and plant processes known to be controlled by them are summarized in Figure 1. The scheme shows how the cytokinin signal splits up into several strands, each one transduced by its own component to result in the different hormonal actions.

One conspicuous observation is that although the percentage of kinase-encoding genes in the Arabidopsis genome is >3%, there was only one cytokinin-regulated (0.05%) kinase found among the set of genes considered as the most reliably cytokinin regulated ones outside of the phosphorelay signaling system. The activity of kinases is usually regulated by means other than their mere abundance, and the transcript levels of genes encoding kinases are often quite stable under a wide range of conditions. Kinases are rather regulated by other means such as the presence of ligands or posttranslational modification. This way, transcriptomic experiments do not necessarily shed light on the kinase parts of signaling pathways, limiting the approach using transcriptomic data in this respect. On the other hand, transcriptomic data are easy to generate and may serve as a starting point for in-depth investigations leading to the discovery of signaling chains involving events other than transcriptional regulation such as kinase activities.

### Feed-Forward and Feed-Back Loops Must Be Spatially and Temporally Separated

It is known that there is an immediate feed-back loop built into the phosphorelay system in the form of the type-A response regulators (Kiba et al., 2003; To et al., 2004; Lee et al., 2007). The existence of feed-back at the level of hormone homeostasis by deactivating and degrading enzymes has also been noted (Kieber and Schaller, 2014). Generally, feed-back loops are a frequently emerging theme in developmental biology, and their roles are exhaustingly covered.

In contrast, the role of a feed-forward mechanism at the level of cytokinin homeostasis has not found much attention. It is generally contradicting the paradigm of the self-limiting action of the hormone in order to maintain stable developmental processes. One scenario that requires escalating a signal by a feed-forward mechanism is rapid long-distance signaling as it happens, for instance, during the propagation of the electrical signal in the axons of nerve cells followed by a delayed feed-back mechanism.

It is certain that cytokinin is the long-distance signal to transmit nitrogen availability to the shoot tip in order to control a sustainable growth rate of the shoot (Miyawaki et al., 2004; Takei et al., 2004a). The cytokinins transported shootward in the xylem belong to the tZ type, the members of which are catalytically formed by the CYP735A enzymes from iP-type cytokinins (Takei et al., 2004b; Kiba et al., 2013). The *CYP735A2* gene is responsive to both nitrate and cytokinin and mainly expressed in the root, but it has not been determined in which cell types it is expressed. It is tempting to speculate that CYP735A2 induced in cells neighboring xylem elements (e.g., xylem parenchyma cells) may lead to a rapid increase of tZ-type cytokinins in the xylem vessels, even more so as the protein is predicted to be localized in the apoplast. The increase of active tZ would in turn trigger the induction of CYP735A2 activity in cells further upstream, releasing more tZ into the xylem in the shootward direction. This mechanism could speed up the migration of the tZ signal beyond the velocity of the xylem stream. Thus, the signal would travel at a speed largely independent of the velocity of the water stream in the xylem vessels, which strongly depends on the transpiration rate, and would be driven by the feed-forward loop of biosynthesis, perception and signaling, rapidly propagating over long distances. To this end, *CYP735A2* should be expected to be expressed in cells neighboring the xylem vessels, such as xylem parenchyma cells. This hypothesis, however, remains to be tested.

Feed-forward and feed-back mechanisms have to be carefully controlled as they may form a wasteful short-circuit if they are active at the same time or place. Thus, it is to be expected to find feed-forward components distinctly from feed-back components. In certain situations, there may be a temporal succession of a feed-forward phase followed by a feed-back phase to first escalate the cytokinin signal before seeking homeostasis.

### Multi-Layered Cytokinin–Auxin Interplay

Auxin and cytokinin action are closely interwoven and each of the two hormone influences the status of the other at multiple layers and through multiple signaling pathways, mostly in an antagonistic fashion (Dello Ioio et al., 2008; Müller and Sheen, 2008). Components of that largely unknown network continue to be discovered, such as *SYAC1* very recently (Hurný et al., 2020).

Some of the cytokinin–auxin crosstalk components, however, have been characterized. Polar auxin transport may be influenced via CRFs transcriptionally regulating PIN expression (Šimášková et al., 2015). The cytokinin-induced MYB-related transcription factor MEE3 is a negative regulator of the HOOKLESS1-dependent auxin signaling pathway during early seedling morphogenesis (Hamaguchi et al., 2008). Cytokinin-stimulated *PILS5* expression may sequester auxin into the ER, removing it from the nucleus where it is supposed to exhibit its activity (Barbez et al., 2012; Feraru et al., 2012; Sun L. et al., 2020). All three examples of crosstalk show a negative influence of cytokinin to auxin action. On the other hand, cytokinin upregulates auxin biosynthesis by increasing *TAA1* and *YUCCA8* expression (Jones et al., 2010; Zhou Z. et al., 2011; Schaller et al., 2015; Di et al., 2016). The crucial function of the two hormones acting in complementary patterns in many developing structures of the plant has been reviewed to great detail (Schaller et al., 2015).

### Downstream Effectors Mediate Part of the Cytokinin Action

Despite the discovery of multiple signaling hubs mediating crosstalk between cytokinin and other pathways for hormone, environmental, and developmental signals, it is still not understood how the multitude of hormonal effects comes into action. Light may be shed on parts of these unknown links by investigating how cytokinin affects the levels and activities of downstream effectors. Cytokinin has been implicated in the regulation of sink–source relationships (Werner et al., 2008; Kieber and Schaller, 2014). The finding that genes responsible for the homeostasis of a master regulator of primary metabolism, trehalose-6-phosphate, are differentially regulated in cytokinin-treated and cytokinin-deficient plants may give a clue on how the cytokinin signal is integrated into the control of primary metabolism.

The general proliferative effect of cytokinin may be mediated by phytosulfokine (PSK), as one of the five PSK precursor genes is positively regulated by the hormone. Not only is PSK regarded as the plant growth factor, but it is also implicated in chloroplast development and other processes driven by cytokinin. Thus, it is tempting to speculate that PSK is a downstream regulator for a significant part of the hormonal action of cytokinin regarding plant development. However, a conclusive loss-of-function experiment is missing due to difficulties obtaining a pertinent mutant.

## Author Contributions

CK selected the genes according to their frequency of occurrence in published transcriptomic studies, collected published information about genes and wrote part of the manuscript. WB initiated the research, supervised the writing process, collected published information about genes, wrote part of the manuscript, including introduction and discussion. Both authors contributed to the article and approved the submitted version.

## Conflict of Interest

The authors declare that the research was conducted in the absence of any commercial or financial relationships that could be construed as a potential conflict of interest.

## References

[B1] Alabadí D. Blázquez M. A. (2008). Integration of light and hormone signals. *Plant Signal. Behav.* 3 448–449. 10.4161/psb.3.7.5558 19704480 PMC2634424

[B2] Alabadıì D. Yanovsky M. J. Más P. Harmer S. L. Kay S. A. (2002). Critical role for CCA1 and LHY in maintaining circadian rhythmicity in *Arabidopsis*. *Curr. Biol.* 12 757–761. 10.1016/S0960-9822(02)00815-1 12007421

[B3] An L. Zhou Z. Sun L. Yan A. Xi W. Yu N. (2012). A zinc finger protein gene ZFP5 integrates phytohormone signaling to control root hair development in *Arabidopsis*. *Plant J.* 72 474–490. 10.1111/j.1365-313X.2012.05094.x 22762888

[B4] Argueso C. T. Ferreira F. J. Kieber J. J. (2009). Environmental perception avenues: the interaction of cytokinin and environmental response pathways. *Plant Cell Environ.* 32 1147–1160. 10.1111/j.1365-3040.2009.01940.x 19183294

[B5] Argyros R. D. Mathews D. E. Chiang Y.-H. Palmer C. M. Thibault D. M. Etheridge N. (2008). Type B response regulators of *Arabidopsis* play key roles in cytokinin signaling and plant development. *Plant Cell* 20 2102–2116. 10.1105/tpc.108.059584 18723577 PMC2553617

[B6] Ascencio-Ibáñez J. T. Sozzani R. Lee T.-J. Chu T.-M. Wolfinger R. D. Cella R. (2008). Global analysis of *Arabidopsis* gene expression uncovers a complex array of changes impacting pathogen response and cell cycle during geminivirus infection. *Plant Physiol.* 148 436–454. 10.1104/pp.108.121038 18650403 PMC2528102

[B7] Barbez E. Kleine-Vehn J. (2013). Divide Et Impera—cellular auxin compartmentalization. *Curr. Opin. Plant Biol.* 16 78–84. 10.1016/j.pbi.2012.10.005 23200033

[B8] Barbez E. Kubeš M. Rolčík J. Béziat C. Pěnčík A. Wang B. (2012). A novel putative auxin carrier family regulates intracellular auxin homeostasis in plants. *Nature* 485 119–122. 10.1038/nature11001 22504182

[B9] Bartrina I. Otto E. Strnad M. Werner T. Schmülling T. (2011). Cytokinin regulates the activity of reproductive meristems, flower organ size, ovule formation, and thus seed yield in *Arabidopsis thaliana*. *Plant Cell* 23 69–80. 10.1105/tpc.110.079079 21224426 PMC3051259

[B10] Bastakis E. Hedtke B. Klermund C. Grimm B. Schwechheimer C. (2018). LLM-Domain B-GATA transcription factors play multifaceted roles in controlling greening in *Arabidopsis*. *Plant Cell* 30 582–599. 10.1105/tpc.17.00947 29453227 PMC5894840

[B11] Baxter C. E. L. Costa M. M. R. Coen E. S. (2007). Diversification and co-option of RAD-like genes in the evolution of floral asymmetry. *Plant J.* 52 105–113. 10.1111/j.1365-313X.2007.03222.x 17672842

[B12] Bezerra-Neto J. P. Czekalski, de Araújo F. Ferreira-Neto J. R. C. da Silva M. D. Pandolfi V. (2019). Plant aquaporins: diversity, evolution and biotechnological applications. *Curr. Protein Pept. Sci.* 20 368–395. 10.2174/1389203720666181102095910 30387391

[B13] Béziat C. Barbez E. Feraru M. I. Lucyshyn D. Kleine-Vehn J. (2017). Light triggers PILS-dependent reduction in nuclear auxin signalling for growth transition. *Nat. Plants* 3:17105. 10.1038/nplants.2017.105 28714973 PMC5524181

[B14] Bhargava A. Clabaugh I. To J. P. Maxwell B. B. Chiang Y.-H. Schaller G. E. (2013). Identification of cytokinin-responsive genes using microarray meta-analysis and RNA-Seq in *Arabidopsis*. *Plant Physiol.* 162 272–294. 10.1104/pp.113.217026 23524861 PMC3641208

[B15] Bi Y.-M. Zhang Y. Signorelli T. Zhao R. Zhu T. Rothstein S. (2005). Genetic analysis of *Arabidopsis* GATA transcription factor gene family reveals a nitrate-inducible member important for chlorophyll synthesis and glucose sensitivity. *Plant J.* 44 680–692. 10.1111/j.1365-313X.2005.02568.x 16262716

[B16] Bielach A. Hrtyan M. Tognetti V. B. (2017). Plants under stress: involvement of auxin and cytokinin. *Int. J. Mol. Sci.* 18:1427. 10.3390/ijms18071427 28677656 PMC5535918

[B17] Bielach A. Podlešáková K. Marhavı P. Duclercq J. Cuesta C. Müller B. (2012). Spatiotemporal regulation of lateral root organogenesis in *Arabidopsis* by cytokinin. *Plant Cell* 24 3967–3981. 10.1105/tpc.112.103044 23054471 PMC3517230

[B18] Bilyeu K. D. Cole J. L. Laskey J. G. Riekhof W. R. Esparza T. J. Kramer M. D. (2001). Molecular and biochemical characterization of a cytokinin oxidase from maize. *Plant Physiol.* 125 378–386. 10.1104/pp.125.1.378 11154345 PMC61018

[B19] Bishopp A. Lehesranta S. Vatén A. Help H. El-Showk S. Scheres B. (2011). Phloem-transported cytokinin regulates polar auxin transport and maintains vascular pattern in the root meristem. *Curr. Biol.* 21 927–932. 10.1016/j.cub.2011.04.049 21620705

[B20] Blom N. Gammeltoft S. Brunak S. (1999). Sequence and structure-based prediction of eukaryotic protein phosphorylation sites1 1edited by F. E. Cohen. *J. Mol. Biol.* 294 1351–1362. 10.1006/jmbi.1999.3310 10600390

[B21] Blom N. Sicheritz-Pontén T. Gupta R. Gammeltoft S. Brunak S. (2004). Prediction of post-translational glycosylation and phosphorylation of proteins from the amino acid sequence. *Proteomics* 4 1633–1649. 10.1002/pmic.200300771 15174133

[B22] Brenner W. G. Leuendorf J. E. Cortleven A. Martin L. B. B. Schaller H. Schmülling T. (2017). Analysis of CFB, a cytokinin-responsive gene of *Arabidopsis thaliana* encoding a novel F-box protein regulating sterol biosynthesis. *J. Exp. Biol.* 68 2769–2785. 10.1093/jxb/erx146 28505379 PMC5853388

[B23] Brenner W. G. Ramireddy E. Heyl A. Schmülling T. (2012). Gene regulation by cytokinin in *Arabidopsis*. *Front. Plant Sci.* 3:8. 10.3389/fpls.2012.00008 22639635 PMC3355611

[B24] Brenner W. G. Romanov G. A. Köllmer I. Bürkle L. Schmülling T. (2005). Immediate-early and delayed cytokinin response genes of *Arabidopsis thaliana* identified by genome-wide expression profiling reveal novel cytokinin-sensitive processes and suggest cytokinin action through transcriptional cascades. *Plant J.* 44 314–333. 10.1111/j.1365-313X.2005.02530.x 16212609

[B25] Brenner W. G. Schmülling T. (2012). Transcript profiling of cytokinin action in *Arabidopsis* roots and shoots discovers largely similar but also organ-specific responses. *BMC Plant Biol.* 12:112. 10.1186/1471-2229-12-112 22824128 PMC3519560

[B26] Brenner W. G. Schmülling T. (2015). Summarizing and exploring data of a decade of cytokinin-related transcriptomics. *Front. Plant Sci.* 6:29. 10.3389/fpls.2015.00029 25741346 PMC4330702

[B27] Caesar K. Thamm A. M. K. Witthöft J. Elgass K. Huppenberger P. Grefen C. (2011). Evidence for the localization of the *Arabidopsis* cytokinin receptors AHK3 and AHK4 in the endoplasmic reticulum. *J. Exp. Bot.* 62 5571–5580. 10.1093/jxb/err238 21841169 PMC3223052

[B28] Causier B. Ashworth M. Guo W. Davies B. (2012). The TOPLESS interactome: a framework for gene repression in *Arabidopsis*. *Plant Physiol.* 158 423–438. 10.1104/pp.111.186999 22065421 PMC3252085

[B29] Chang G. Wang C. Kong X. Chen Q. Yang Y. Hu X. (2018). AFP2 as the novel regulator breaks high-temperature-induced seeds secondary dormancy through ABI5 and SOM in *Arabidopsis thaliana*. *Biochem. Biophys. Res. Commun.* 501 232–238. 10.1016/j.bbrc.2018.04.222 29723526

[B30] Chang G. Yang W. Zhang Q. Huang J. Yang Y. Hu X. (2019). ABI5-BINDING PROTEIN2 coordinates CONSTANS to delay flowering by recruiting the transcriptional corepressor TPR2. *Plant Physiol.* 179 477–490. 10.1104/pp.18.00865 30514725 PMC6426417

[B31] Chang L. Ramireddy E. Schmülling T. (2015). Cytokinin as a positional cue regulating lateral root spacing in *Arabidopsis*. *J. Exp. Bot.* 66 4759–4768. 10.1093/jxb/erv252 26019251 PMC4507779

[B32] Che P. Gingerich D. J. Lall S. Howell S. H. (2002). Global and hormone-induced gene expression changes during shoot development in *Arabidopsis*. *Plant Cell* 14 2771–2785. 10.1105/tpc.006668 12417700 PMC152726

[B33] Chen Y. F. Matsubayashi Y. Sakagami Y. (2000). Peptide growth factor phytosulfokine-α contributes to the pollen population effect. *Planta* 211 752–755. 10.1007/s004250000370 11089690

[B34] Chiang Y.-H. Zubo Y. O. Tapken W. Kim H. J. Lavanway A. M. Howard L. (2012). Functional characterization of the GATA transcription factors GNC and CGA1 reveals their key role in chloroplast development, growth, and division in *Arabidopsis*. *Plant Physiol.* 160 332–348. 10.1104/pp.112.198705 22811435 PMC3440210

[B35] Chini A. Fonseca S. Fernández G. Adie B. Chico J. M. Lorenzo O. (2007). The JAZ family of repressors is the missing link in jasmonate signalling. *Nature* 448 666–671. 10.1038/nature06006 17637675

[B36] Ckurshumova W. Smirnova T. Marcos D. Zayed Y. Berleth T. (2014). Irrepressible MONOPTEROS/ARF5 promotes de novo shoot formation. *New Phytol.* 204 556–566. 10.1111/nph.13014 25274430

[B37] Corbesier L. Prinsen E. Jacqmard A. Lejeune P. Van Onckelen H. Périlleux C. (2003). Cytokinin levels in leaves, leaf exudate and shoot apical meristem of Arabidopsis thaliana during floral transition. *J. Exp. Bot.* 54 2511–2517. 10.1093/jxb/erg276 14512385

[B38] Cortleven A. Leuendorf J. E. Frank M. Pezzetta D. Bolt S. Schmülling T. (2019). Cytokinin action in response to abiotic and biotic stresses in plants. *Plant Cell Environ.* 42 998–1018. 10.1111/pce.13494 30488464

[B39] Cutcliffe J. W. Hellmann E. Heyl A. Rashotte A. M. (2011). CRFs form protein–protein interactions with each other and with members of the cytokinin signalling pathway in *Arabidopsis* via the CRF domain. *J. Exp. Bot.* 62 4995–5002. 10.1093/jxb/err199 21705390 PMC3193008

[B40] Dal Bosco C. Dovzhenko A. Liu X. Woerner N. Rensch T. Eismann M. (2012). The endoplasmic reticulum localized PIN8 is a pollen-specific auxin carrier involved in intracellular auxin homeostasis. *Plant J.* 71 860–870. 10.1111/j.1365-313X.2012.05037.x 22540348

[B41] Dello Ioio R. Nakamura K. Moubayidin L. Perilli S. Taniguchi M. Morita M. T. (2008). A genetic framework for the control of cell division and differentiation in the root meristem. *Science* 322 1380–1384. 10.1126/science.1164147 19039136

[B42] Di D.-W. Wu L. Zhang L. An C.-W. Zhang T.-Z. Luo P. (2016). Functional roles of *Arabidopsis* CKRC2/YUCCA8 gene and the involvement of PIF4 in the regulation of auxin biosynthesis by cytokinin. *Sci. Rep.* 6:36866. 10.1038/srep36866 27827441 PMC5101810

[B43] Etchells J. P. Provost C. M. Mishra L. Turner S. R. (2013). *WOX4* and *WOX14* act downstream of the PXY receptor kinase to regulate plant vascular proliferation independently of any role in vascular organisation. *Development* 140 2224–2234. 10.1242/dev.091314 23578929 PMC3912870

[B44] Feraru E. Feraru M. I. Barbez E. Waidmann S. Sun L. Gaidora A. (2019). PILS6 is a temperature-sensitive regulator of nuclear auxin input and organ growth in *Arabidopsis thaliana*. *Proc. Natl. Acad. Sci. U.S.A.* 116 3893–3898. 10.1073/pnas.1814015116 30755525 PMC6397578

[B45] Feraru E. Vosolsobì S. Feraru M. Petrášek J. Kleine-Vehn J. (2012). Evolution and structural diversification of PILS putative auxin carriers in plants. *Front. Plant Sci.* 3:227. 10.3389/fpls.2012.00227 23091477 PMC3470039

[B46] Fernandez-Calvino L. Faulkner C. Walshaw J. Saalbach G. Bayer E. Benitez-Alfonso Y. (2011). *Arabidopsis* plasmodesmal proteome. *PLoS One* 6:e18880. 10.1371/journal.pone.0018880 21533090 PMC3080382

[B47] Fichtner F. Olas J. J. Feil R. Watanabe M. Krause U. Hoefgen R. (2020). Functional features of TREHALOSE-6-PHOSPHATE SYNTHASE1, an essential enzyme in *Arabidopsis*. *Plant Cell* 32 1949–1972. 10.1105/tpc.19.00837 32276986 PMC7268806

[B48] Figueroa C. M. Lunn J. E. (2016). A tale of two sugars: Trehalose 6-Phosphate and sucrose. *Plant Physiol.* 172 7–27. 10.1104/pp.16.00417 27482078 PMC5074632

[B49] Finkelstein R. R. (1994). Maternal effects govern variable dominance of two abscisic acid response mutations in *Arabidopsis thaliana*. *Plant Physiol.* 105 1203–1208. 10.1104/pp.105.4.1203 12232276 PMC159449

[B50] Finkelstein R. R. Lynch T. J. (2000). The *Arabidopsis* abscisic acid response gene *ABI5* encodes a basic leucine zipper transcription factor. *Plant Cell* 12 599–609. 10.1105/tpc.12.4.599 10760247 PMC139856

[B51] Franco-Zorrilla J. M. López-Vidriero I. Carrasco J. L. Godoy M. Vera P. Solano R. (2014). DNA-binding specificities of plant transcription factors and their potential to define target genes. *Proc. Natl. Acad. Sci. U.S.A.* 111 2367–2372. 10.1073/pnas.1316278111 24477691 PMC3926073

[B52] Gajdošová S. Spíchal L. Kamínek M. Hoyerová K. Novák O. Dobrev P. I. (2011). Distribution, biological activities, metabolism, and the conceivable function of cis-zeatin-type cytokinins in plants. *J. Exp. Bot.* 62 2827–2840. 10.1093/jxb/erq457 21282330

[B53] Gan S. Amasino R. M. (1995). Inhibition of leaf senescence by autoregulated production of cytokinin. *Science* 270 1986–1988. 10.1126/science.270.5244.1986 8592746

[B54] Gan Y. Liu C. Yu H. Broun P. (2007a). Integration of cytokinin and gibberellin signalling by *Arabidopsis* transcription factors GIS, ZFP8 and GIS2 in the regulation of epidermal cell fate. *Development* 134 2073–2081. 10.1242/dev.005017 17507408

[B55] Gan Y. Yu H. Peng J. Broun P. (2007b). Genetic and molecular regulation by DELLA proteins of trichome development in *Arabidopsis*. *Plant Physiol.* 145 1031–1042. 10.1104/pp.107.104794 17704233 PMC2048772

[B56] Garcia M. E. Lynch T. Peeters J. Snowden C. Finkelstein R. (2008). A small plant-specific protein family of ABI five binding proteins (AFPs) regulates stress response in germinating *Arabidopsis* seeds and seedlings. *Plant Mol. Biol.* 67 643–658. 10.1007/s11103-008-9344-2 18484180

[B57] Gaudet P. Livstone M. S. Lewis S. E. Thomas P. D. (2011). Phylogenetic-based propagation of functional annotations within the gene ontology consortium. *Brief. Bioinform.* 12 449–462. 10.1093/bib/bbr042 21873635 PMC3178059

[B58] Greenboim-Wainberg Y. Maymon I. Borochov R. Alvarez J. Olszewski N. Ori N. (2005). Cross talk between gibberellin and cytokinin: the *Arabidopsis* GA response inhibitor SPINDLY plays a positive role in cytokinin signaling. *Plant Cell* 17 92–102. 10.1105/tpc.104.028472 15608330 PMC544492

[B59] Hamaguchi A. Yamashino T. Koizumi N. Kiba T. Kojima M. Sakakibara H. (2008). A small subfamily of *Arabidopsis* RADIALIS-LIKE SANT/MYB genes: a link to HOOKLESS1-mediated signal transduction during early morphogenesis. *Biosci. Biotechnol. Biochem.* 72 2687–2696. 10.1271/bbb.80348 18838801

[B60] Han G. Lu C. Guo J. Qiao Z. Sui N. Qiu N. (2020). C2H2 Zinc finger proteins: master regulators of abiotic stress responses in plants. *Front. Plant Sci.* 11:115. 10.3389/fpls.2020.00115 32153617 PMC7044346

[B61] Hanai H. Matsuno T. Yamamoto M. Matsubayashi Y. Kobayashi T. Kamada H. (2000). A secreted peptide growth factor, phytosulfokine, acting as a stimulatory factor of carrot somatic embryo formation. *Plant Cell Physiol.* 41 27–32. 10.1093/pcp/41.1.27 10750705

[B62] Hao D. Ohme-Takagi M. Sarai A. (1998). Unique mode of GCC box recognition by the DNA-binding domain of ethylene-responsive element-binding factor (ERF domain) in plant. *J. Biol. Chem.* 273 26857–26861. 10.1074/jbc.273.41.26857 9756931

[B63] Harmer S. L. Hogenesch J. B. Straume M. Chang H.-S. Han B. Zhu T. (2000). Orchestrated transcription of key pathways in *Arabidopsis* by the circadian clock. *Science* 290 2110–2113. 10.1126/science.290.5499.2110 11118138

[B64] Héricourt F. Chefdor F. Bertheau L. Tanigawa M. Maeda T. Guirimand G. (2013). Characterization of histidine-aspartate kinase HK1 and identification of histidine phosphotransfer proteins as potential partners in a *Populus* multistep phosphorelay. *Physiol. Plant.* 149 188–199. 10.1111/ppl.12024 23330606

[B65] Héricourt F. Chefdor F. Djeghdir I. Larcher M. Lafontaine F. Courdavault V. (2016). Functional divergence of poplar histidine-aspartate kinase HK1 paralogs in response to osmotic stress. *Int. J. Mol. Sci.* 17:2061. 10.3390/ijms17122061 27941652 PMC5187861

[B66] Héricourt F. Larcher M. Chefdor F. Koudounas K. Carqueijeiro I. Lemos Cruz P. (2019). New insight into HPts as hubs in poplar cytokinin and osmosensing multistep phosphorelays: cytokinin pathway uses specific HPts. *Plants* 8:591. 10.3390/plants8120591 31835814 PMC6963366

[B67] Heyl A. Brault M. Frugier F. Kuderova A. Lindner A.-C. Motyka V. (2013). Nomenclature for members of the two-component signaling pathway of plants. *Plant Physiol.* 161 1063–1065. 10.1104/pp.112.213207 23324541 PMC3585578

[B68] Heyl A. Ramireddy E. Brenner W. G. Riefler M. Allemeersch J. Schmülling T. (2008). The transcriptional repressor ARR1-SRDX suppresses pleiotropic cytokinin activities in *Arabidopsis*. *Plant Physiol.* 147 1380–1395. 10.1104/pp.107.115436 18502977 PMC2442517

[B69] Hirakawa Y. Kondo Y. Fukuda H. (2010). TDIF peptide signaling regulates vascular stem cell proliferation via the *WOX4* homeobox gene in *Arabidopsis*. *Plant Cell* 22 2618–2629. 10.1105/tpc.110.076083 20729381 PMC2947162

[B70] Hirakawa Y. Shinohara H. Kondo Y. Inoue A. Nakanomyo I. Ogawa M. (2008). Non-cell-autonomous control of vascular stem cell fate by a CLE peptide/receptor system. *Proc. Natl. Acad. Sci. U.S.A.* 105 15208–15213. 10.1073/pnas.0808444105 18812507 PMC2567516

[B71] Hirose N. Takei K. Kuroha T. Kamada-Nobusada T. Hayashi H. Sakakibara H. (2007). Regulation of cytokinin biosynthesis, compartmentalization and translocation. *J. Exp. Bot.* 59 75–83. 10.1093/jxb/erm157 17872922

[B72] Hoth S. Ikeda Y. Morgante M. Wang X. Zuo J. Hanafey M. K. (2003). Monitoring genome-wide changes in gene expression in response to endogenous cytokinin reveals targets in *Arabidopsis thaliana*. *FEBS Lett.* 554 373–380. 10.1016/s0014-5793(03)01194-3 14623097

[B73] Huang X. Hou L. Meng J. You H. Li Z. Gong Z. (2018). The antagonistic action of abscisic acid and cytokinin signaling mediates drought stress response in *Arabidopsis*. *Mol. Plant* 11 970–982. 10.1016/j.molp.2018.05.001 29753021

[B74] Hudson D. Guevara D. Yaish M. W. Hannam C. Long N. Clarke J. D. (2011). GNC and CGA1 modulate chlorophyll biosynthesis and glutamate synthase (GLU1/Fd-GOGAT) expression in *Arabidopsis*. *PLoS One* 6:e26765. 10.1371/journal.pone.0026765 22102866 PMC3213100

[B75] Hudson M. E. Lisch D. R. Quail P. H. (2003). The FHY3 and FAR1 genes encode transposase-related proteins involved in regulation of gene expression by the phytochrome A-signaling pathway. *Plant J.* 34 453–471. 10.1046/j.1365-313x.2003.01741.x 12753585

[B76] Hurný A. Cuesta C. Cavallari N. Ötvös K. Duclercq J. Dokládal L. (2020). SYNERGISTIC ON AUXIN AND CYTOKININ 1 positively regulates growth and attenuates soil pathogen resistance. *Nat. Commun.* 11:2170. 10.1038/s41467-020-15895-5 32358503 PMC7195429

[B77] Igasaki T. Akashi N. Ujino-Ihara T. Matsubayashi Y. Sakagami Y. Shinohara K. (2003). Phytosulfokine stimulates somatic embryogenesis in *Cryptomeria japonica*. *Plant Cell Physiol.* 44 1412–1416. 10.1093/pcp/pcg161 14701937

[B78] Iwakawa H. Ueno Y. Semiarti E. Onouchi H. Kojima S. Tsukaya H. (2002). The *ASYMMETRIC LEAVES2* gene of *Arabidopsis thaliana*, required for formation of a symmetric flat leaf lamina, encodes a member of a novel family of proteins characterized by cysteine repeats and a leucine zipper. *Plant Cell Physiol.* 43 467–478. 10.1093/pcp/pcf077 12040093

[B79] Jeon J. Cho C. Lee M. R. Van Binh N. Kim J. (2016). CYTOKININ RESPONSE FACTOR2 (CRF2) and CRF3 regulate lateral root development in response to cold stress in *Arabidopsis*. *Plant Cell* 28 1828–1843. 10.1105/tpc.15.00909 27432872 PMC5006697

[B80] Jones B. Gunnerås S. A. Petersson S. V. Tarkowski P. Graham N. May S. (2010). Cytokinin regulation of auxin synthesis in *Arabidopsis* involves a homeostatic feedback loop regulated via auxin and cytokinin signal transduction. *Plant Cell* 22 2956–2969. 10.1105/tpc.110.074856 20823193 PMC2965550

[B81] Kiba T. Aoki K. Sakakibara H. Mizuno T. (2004). Arabidopsis response regulator, ARR22, ectopic expression of which results in phenotypes similar to the wol cytokinin-receptor mutant. *Plant Cell Physiol.* 45 1063–1077. 10.1093/pcp/pch128 15356332

[B82] Kiba T. Naitou T. Koizumi N. Yamashino T. Sakakibara H. Mizuno T. (2005). Combinatorial microarray analysis revealing *Arabidopsis* genes implicated in cytokinin responses through the His→Asp phosphorelay circuitry. *Plant Cell Physiol.* 46 339–355. 10.1093/pcp/pci033 15695462

[B83] Kiba T. Takei K. Kojima M. Sakakibara H. (2013). Side-chain modification of cytokinins controls shoot growth in *Arabidopsis*. *Dev. Cell* 27 452–461. 10.1016/j.devcel.2013.10.004 24286826

[B84] Kiba T. Yamada H. Sato S. Kato T. Tabata S. Yamashino T. (2003). The Type-A response regulator, ARR15, acts as a negative regulator in the cytokinin-mediated signal transduction in *Arabidopsis thaliana*. *Plant Cell Physiol.* 44 868–874. 10.1093/pcp/pcg108 12941880

[B85] Kieber J. J. Schaller G. E. (2014). Cytokinins. *Arabid. Book* 12:e0168. 10.1199/tab.0168 24465173 PMC3894907

[B86] Kieber J. J. Schaller G. E. (2018). Cytokinin signaling in plant development. *Development* 145:dev149344. 10.1242/dev.149344 29487105

[B87] Kim H. J. Ryu H. Hong S. H. Woo H. R. Lim P. O. Lee I. C. (2006). Cytokinin-mediated control of leaf longevity by AHK3 through phosphorylation of ARR2 in *Arabidopsis*. *Proc. Natl. Acad. Sci. U.S.A.* 103 814–819. 10.1073/pnas.0505150103 16407152 PMC1334631

[B88] Klepikova A. V. Kasianov A. S. Gerasimov E. S. Logacheva M. D. Penin A. A. (2016). A high resolution map of the *Arabidopsis thaliana* developmental transcriptome based on RNA-seq profiling. *Plant J.* 88 1058–1070. 10.1111/tpj.13312 27549386

[B89] Kobayashi K. Iwase A. (2017). Simultaneous but spatially different regulation of non-photosynthetic callus formation and photosynthetic root development after shoot removal. *Plant Signal. Behav.* 12:e1338999. 10.1080/15592324.2017.1338999 28594268 PMC5566382

[B90] Kobayashi K. Ohnishi A. Sasaki D. Fujii S. Iwase A. Sugimoto K. (2017). Shoot removal induces chloroplast development in roots via cytokinin signaling. *Plant Physiol.* 173 2340–2355. 10.1104/pp.16.01368 28193764 PMC5373043

[B91] Kobayashi T. Eun C.-H. Hanai H. Matsubayashi Y. Sakagami Y. Kamada H. (1999). Phytosulphokine-α, a peptidyl plant growth factor, stimulates somatic embryogenesis in carrot. *J. Exp. Bot.* 50 1123–1128. 10.1093/jxb/50.336.1123 12432039

[B92] Köllmer I. Werner T. Schmülling T. (2011). Ectopic expression of different cytokinin-regulated transcription factor genes of *Arabidopsis thaliana* alters plant growth and development. *J. Plant Physiol.* 168 1320–1327. 10.1016/j.jplph.2011.02.006 21453984

[B93] Kondo Y. Ito T. Nakagami H. Hirakawa Y. Saito M. Tamaki T. (2014). Plant GSK3 proteins regulate xylem cell differentiation downstream of TDIF–TDR signalling. *Nat. Commun.* 5:3504. 10.1038/ncomms4504 24662460

[B94] Kowalska M. Galuszka P. Frébortová J. Šebela M. Béres T. Hluska T. (2010). Vacuolar and cytosolic cytokinin dehydrogenases of *Arabidopsis thaliana*: heterologous expression, purification and properties. *Phytochemistry* 71 1970–1978. 10.1016/j.phytochem.2010.08.013 20825956

[B95] Krouk G. Ruffel S. Gutiérrez R. A. Gojon A. Crawford N. M. Coruzzi G. M. (2011). A framework integrating plant growth with hormones and nutrients. *Trends Plant Sci.* 16 178–182. 10.1016/j.tplants.2011.02.004 21393048

[B96] Kuderová A. Urbánková I. Válková M. Malbeck J. Brzobohatı B. Némethová D. (2008). Effects of conditional IPT-dependent cytokinin overproduction on root architecture of *Arabidopsis* seedlings. *Plant Cell Physiol.* 49 570–582. 10.1093/pcp/pcn029 18296451

[B97] Kudo T. Kiba T. Sakakibara H. (2010). Metabolism and long-distance translocation of cytokinins. *J. Integ. Plant Biol.* 52 53–60. 10.1111/j.1744-7909.2010.00898.x 20074140

[B98] Kumar M. N. Jane W.-N. Verslues P. E. (2013). Role of the putative osmosensor *Arabidopsis* Histidine Kinase1 in dehydration avoidance and low-water-potential response. *Plant Physiol.* 161 942–953. 10.1104/pp.112.209791 23184230 PMC3561031

[B99] Laplaze L. Benkova E. Casimiro I. Maes L. Vanneste S. Swarup R. (2007). Cytokinins act directly on lateral root founder cells to inhibit root initiation. *Plant Cell* 19 3889–3900. 10.1105/tpc.107.055863 18065686 PMC2217640

[B100] Lee D. J. Park J.-Y. Ku S.-J. Ha Y.-M. Kim S. Kim M. D. (2007). Genome-wide expression profiling of ARABIDOPSIS RESPONSE REGULATOR 7(ARR7) overexpression in cytokinin response. *Mol. Genet. Genomics* 277 115–137. 10.1007/s00438-006-0177-x 17061125

[B101] Li L. He Z. Pandey G. K. Tsuchiya T. Luan S. (2002). Functional cloning and characterization of a plant efflux carrier for multidrug and heavy metal detoxification. *J. Biol. Chem.* 277 5360–5368. 10.1074/jbc.M108777200 11739388

[B102] Li Y. J. Wang B. Dong R. R. Hou B. K. (2015). AtUGT76C2, an *Arabidopsis* cytokinin glycosyltransferase is involved in drought stress adaptation. *Plant Sci.* 236 157–167. 10.1016/j.plantsci.2015.04.002 26025529

[B103] Lopez-Molina L. Mongrand S. Chua N.-H. (2001). A postgermination developmental arrest checkpoint is mediated by abscisic acid and requires the ABI5 transcription factor in *Arabidopsis*. *Proc. Natl. Acad. Sci. U.S.A.* 98 4782–4787. 10.1073/pnas.081594298 11287670 PMC31911

[B104] Lopez-Molina L. Mongrand S. Kinoshita N. Chua N.-H. (2003). AFP is a novel negative regulator of ABA signaling that promotes ABI5 protein degradation. *Genes Dev.* 17 410–418. 10.1101/gad.1055803 12569131 PMC195991

[B105] Lynch T. J. Erickson B. J. Miller D. R. Finkelstein R. R. (2017). ABI5-binding proteins (AFPs) alter transcription of ABA-induced genes via a variety of interactions with chromatin modifiers. *Plant Mol. Biol.* 93 403–418. 10.1007/s11103-016-0569-1 27942958

[B106] Manfield I. W. Devlin P. F. Jen C.-H. Westhead D. R. Gilmartin P. M. (2007). Conservation, convergence, and divergence of light-responsive, circadian-regulated, and tissue-specific expression patterns during evolution of the *Arabidopsis* GATA gene family. *Plant Physiol.* 143 941–958. 10.1104/pp.106.090761 17208962 PMC1803723

[B107] Mara C. D. Irish V. F. (2008). Two GATA transcription factors are downstream effectors of floral homeotic gene action in *Arabidopsis*. *Plant Physiol.* 147 707–718. 10.1104/pp.107.115634 18417639 PMC2409029

[B108] Matsubayashi Y. Ogawa M. Kihara H. Niwa M. Sakagami Y. (2006a). Disruption and overexpression of *Arabidopsis* phytosulfokine receptor gene affects cellular longevity and potential for growth. *Plant Physiol.* 142 45–53. 10.1104/pp.106.081109 16829587 PMC1557600

[B109] Matsubayashi Y. Shinohara H. Ogawa M. (2006b). Identification and functional characterization of phytosulfokine receptor using a ligand-based approach. *Chem. Rec.* 6 356–364. 10.1002/tcr.20090 17304545

[B110] Matsubayashi Y. Takagi L. Omura N. Morita A. Sakagami Y. (1999). The endogenous sulfated pentapeptide phytosulfokine-α stimulates tracheary element differentiation of isolated mesophyll cells of zinnia. *Plant Physiol.* 120 1043–1048. 10.1104/pp.120.4.1043 10444087 PMC59337

[B111] Matsumoto-Kitano M. Kusumoto T. Tarkowski P. Kinoshita-Tsujimura K. Václavíková K. Miyawaki K. (2008). Cytokinins are central regulators of cambial activity. *Proc. Natl. Acad. Sci. U.S.A.* 105 20027–20031. 10.1073/pnas.0805619105 19074290 PMC2605004

[B112] Menges M. Hennig L. Gruissem W. Murray J. A. H. (2002). Cell cycle-regulated gene expression in *Arabidopsis*. *J. Biol. Chem.* 277 41987–42002. 10.1074/jbc.M207570200 12169696

[B113] Miyawaki K. Matsumoto-Kitano M. Kakimoto T. (2004). Expression of cytokinin biosynthetic isopentenyltransferase genes in *Arabidopsis*: tissue specificity and regulation by auxin, cytokinin, and nitrate. *Plant J.* 37 128–138. 10.1046/j.1365-313X.2003.01945.x 14675438

[B114] Mok D. W. Mok M. C. (2001). Cytokinin metabolism and action. *Annu. Rev. Plant Physiol. Plant Mol. Biol.* 52 89–118. 10.1146/annurev.arplant.52.1.89 11337393

[B115] Mok M. C. Mok D. W. S. Armstrong D. J. (1978). Differential cytokinin structure-activity relationships in *Phaseolus*. *Plant Physiol.* 61 72–75. 10.1104/pp.61.1.72 16660241 PMC1091799

[B116] Mori K. Renhu N. Naito M. Nakamura A. Shiba H. Yamamoto T. (2018). Ca2+-permeable mechanosensitive channels MCA1 and MCA2 mediate cold-induced cytosolic Ca2+ increase and cold tolerance in *Arabidopsis*. *Sci. Rep.* 8:550. 10.1038/s41598-017-17483-y 29323146 PMC5765038

[B117] Morita J. Kato K. Nakane T. Kondo Y. Fukuda H. Nishimasu H. (2016). Crystal structure of the plant receptor-like kinase TDR in complex with the TDIF peptide. *Nat. Commun.* 7:12383. 10.1038/ncomms12383 27498761 PMC4979064

[B118] Müller B. Sheen J. (2007). Advances in cytokinin signaling. *Science* 318 68–69. 10.1126/science.1145461 17916725

[B119] Müller B. Sheen J. (2008). Cytokinin and auxin interaction in root stem-cell specification during early embryogenesis. *Nature* 453 1094–1097. 10.1038/nature06943 18463635 PMC2601652

[B120] Müller M. Munné-Bosch S. (2015). Ethylene response factors: a key regulatory hub in hormone and stress signaling. *Plant Physiol.* 169 32–41. 10.1104/pp.15.00677 26103991 PMC4577411

[B121] Naito T. Yamashino T. Kiba T. Koizumi N. Kojima M. Sakakibara H. (2007). A link between cytokinin and ASL9 (ASYMMETRIC LEAVES 2 LIKE 9) that belongs to the AS2/LOB (LATERAL ORGAN BOUNDARIES) family genes in *Arabidopsis thaliana*. *Biosci. Biotechnol. Biochem.* 71 1269–1278. 10.1271/bbb.60681 17485849

[B122] Nakamura S. Lynch T. J. Finkelstein R. R. (2001). Physical interactions between ABA response loci of *Arabidopsis*. *Plant J.* 26 627–635. 10.1046/j.1365-313x.2001.01069.x 11489176

[B123] Naseem M. Dandekar T. (2012). The role of auxin-cytokinin antagonism in plant-pathogen interactions. *PLoS Pathog.* 8:e1003026. 10.1371/journal.ppat.1003026 23209407 PMC3510258

[B124] Naseem M. Kunz M. Dandekar T. (2014). Probing the unknowns in cytokinin-mediated immune defense in *Arabidopsis* with systems biology approaches. *Bioinform. Biol. Insights* 8 35–44. 10.4137/bbi.S13462 24558299 PMC3929428

[B125] Obulareddy N. Panchal S. Melotto M. (2013). Guard cell purification and RNA isolation suitable for high-throughput transcriptional analysis of cell-type responses to biotic stresses. *Mol. Plant Microbe Interact.* 26 844–849. 10.1094/MPMI-03-13-0081-TA 23634837 PMC3982617

[B126] Pagnussat G. C. Yu H.-J. Ngo Q. A. Rajani S. Mayalagu S. Johnson C. S. (2005). Genetic and molecular identification of genes required for female gametophyte development and function in *Arabidopsis*. *Development* 132 603–614. 10.1242/dev.01595 15634699

[B127] Panstruga R. Baumgarten K. Bernhagen J. (2015). Phylogeny and evolution of plant macrophage migration inhibitory factor/D-dopachrome tautomerase-like proteins. *BMC Evol. Biol.* 15:64. 10.1186/s12862-015-0337-x 25888527 PMC4407349

[B128] Pauwels L. Barbero G. F. Geerinck J. Tilleman S. Grunewald W. Pérez A. C. (2010). NINJA connects the co-repressor TOPLESS to jasmonate signalling. *Nature* 464 788–791. 10.1038/nature08854 20360743 PMC2849182

[B129] Poitout A. Crabos A. Petøík I. Novák O. Krouk G. Lacombe B. (2018). Responses to systemic nitrogen signaling in *Arabidopsis* roots involve *trans*-Zeatin in shoots. *Plant Cell* 30 1243–1257. 10.1105/tpc.18.00011 29764985 PMC6048791

[B130] Price J. Laxmi A. St. Martin S. K. Jang J.-C. (2004). Global transcription profiling reveals multiple sugar signal transduction mechanisms in *Arabidopsis*. *Plant Cell* 16 2128–2150. 10.1105/tpc.104.022616 15273295 PMC519203

[B131] Ramireddy E. Chang L. Schmülling T. (2014). Cytokinin as a mediator for regulating root system architecture in response to environmental cues. *Plant Signal. Behav.* 9:e27771. 10.4161/psb.27771 24509549 PMC4091237

[B132] Ranftl Q. L. Bastakis E. Klermund C. Schwechheimer C. (2016). LLM-domain containing B-GATA factors control different aspects of cytokinin-regulated development in *Arabidopsis thaliana*. *Plant Physiol.* 170 2295–2311. 10.1104/pp.15.01556 26829982 PMC4825128

[B133] Rashotte A. M. Carson S. D. B. To J. P. C. Kieber J. J. (2003). Expression profiling of cytokinin action in *Arabidopsis*. *Plant Physiol.* 132 1998–2011. 10.1104/pp.103.021436 12913156 PMC181285

[B134] Rashotte A. M. Goertzen L. R. (2010). The CRF domain defines cytokinin response factor proteins in plants. *BMC Plant Biol.* 10:74. 10.1186/1471-2229-10-74 20420680 PMC3095348

[B135] Rashotte A. M. Mason M. G. Hutchison C. E. Ferreira F. J. Schaller G. E. Kieber J. J. (2006). A subset of *Arabidopsis* AP2 transcription factors mediates cytokinin responses in concert with a two-component pathway. *Proc. Natl. Acad. Sci. U.S.A.* 103 11081–11085. 10.1073/pnas.0602038103 16832061 PMC1544176

[B136] Reumann S. Babujee L. Ma C. Wienkoop S. Siemsen T. Antonicelli G. E. (2007). Proteome analysis of *Arabidopsis* leaf peroxisomes reveals novel targeting peptides, metabolic pathways, and defense mechanisms. *Plant Cell* 19 3170–3193. 10.1105/tpc.107.050989 17951448 PMC2174697

[B137] Reyes J. C. Muro-Pastor M. I. Florencio F. J. (2004). The GATA family of transcription factors in *Arabidopsis* and rice. *Plant Physiol.* 134 1718–1732. 10.1104/pp.103.037788 15084732 PMC419845

[B138] Richter R. Bastakis E. Schwechheimer C. (2013a). Cross-repressive interactions between SOC1 and the GATAs GNC and GNL/CGA1 in the control of greening, cold tolerance, and flowering time in *Arabidopsis*. *Plant Physiol.* 162 1992–2004. 10.1104/pp.113.219238 23739688 PMC3729777

[B139] Richter R. Behringer C. Zourelidou M. Schwechheimer C. (2013b). Convergence of auxin and gibberellin signaling on the regulation of the GATA transcription factors *GNC* and *GNL* in *Arabidopsis thaliana*. *Proc. Natl. Acad. Sci. U.S.A.* 110 13192–13197. 10.1073/pnas.1304250110 23878229 PMC3740866

[B140] Richter R. Behringer C. Müller I. K. Schwechheimer C. (2010). The GATA-type transcription factors GNC and GNL/CGA1 repress gibberellin signaling downstream from DELLA proteins and phytochrome-interacting factors. *Genes Dev.* 24 2093–2104. 10.1101/gad.594910 20844019 PMC2939370

[B141] Riechmann J. L. Ratcliffe O. J. (2000). A genomic perspective on plant transcription factors. *Curr. Opin. Plant Biol.* 3 423–434. 10.1016/s1369-5266(00)00107-2 11019812

[B142] Riefler M. Novak O. Strnad M. Schmülling T. (2006). *Arabidopsis* cytokinin receptor mutants reveal functions in shoot growth, leaf senescence, seed size, germination, root development, and cytokinin metabolism. *Plant Cell* 18 40–54. 10.1105/tpc.105.037796 16361392 PMC1323483

[B143] Ruffel S. Krouk G. Ristova D. Shasha D. Birnbaum K. D. Coruzzi G. M. (2011). Nitrogen economics of root foraging: transitive closure of the nitrate–cytokinin relay and distinct systemic signaling for N supply vs. demand. *Proc. Natl. Acad. Sci. U.S.A.* 108 18524–18529. 10.1073/pnas.1108684108 22025711 PMC3215050

[B144] Ruffel S. Poitout A. Krouk G. Coruzzi G. M. Lacombe B. (2016). Long-distance nitrate signaling displays cytokinin dependent and independent branches. *J. Integ. Plant Biol.* 58 226–229. 10.1111/jipb.12453 26619818

[B145] Sakuma Y. Liu Q. Dubouzet J. G. Abe H. Shinozaki K. Yamaguchi-Shinozaki K. (2002). DNA-binding specificity of the ERF/AP2 domain of *Arabidopsis* DREBs, transcription factors involved in dehydration- and cold-inducible gene expression. *Biochem. Biophys. Res. Commun.* 290 998–1009. 10.1006/bbrc.2001.6299 11798174

[B146] Sasaki E. Ogura T. Takei K. Kojima M. Kitahata N. Sakakibara H. (2013). Uniconazole, a cytochrome P450 inhibitor, inhibits trans-zeatin biosynthesis in *Arabidopsis*. *Phytochemistry* 87 30–38. 10.1016/j.phytochem.2012.11.023 23280040

[B147] Schaller G. E. Bishopp A. Kieber J. J. (2015). The Yin-Yang of hormones: cytokinin and auxin interactions in plant development. *Plant Cell* 27 44–63. 10.1105/tpc.114.133595 25604447 PMC4330578

[B148] Scheible W.-R. Morcuende R. Czechowski T. Fritz C. Osuna D. Palacios-Rojas N. (2004). Genome-wide reprogramming of primary and secondary metabolism, protein synthesis, cellular growth processes, and the regulatory infrastructure of *Arabidopsis* in response to nitrogen. *Plant Physiol.* 136 2483–2499. 10.1104/pp.104.047019 15375205 PMC523316

[B149] Schlereth A. Möller B. Liu W. Kientz M. Flipse J. Rademacher E. H. (2010). MONOPTEROS controls embryonic root initiation by regulating a mobile transcription factor. *Nature* 464 913–916. 10.1038/nature08836 20220754

[B150] Schmid M. Davison T. S. Henz S. R. Pape U. J. Demar M. Vingron M. (2005). A gene expression map of *Arabidopsis thaliana* development. *Nat. Genet.* 37 501–506. 10.1038/ng1543 15806101

[B151] Schmitz R. Y. Skoog F. Playtis A. J. Leonard N. J. (1972). Cytokinins: synthesis and biological activity of geometric and position isomers of zeatin. *Plant Physiol.* 50 702–705. 10.1104/pp.50.6.702 16658247 PMC366220

[B152] Schmülling T. Werner T. Riefler M. Krupková E. Bartrina y Manns I. (2003). Structure and function of cytokinin oxidase/dehydrogenase genes of maize, rice, *Arabidopsis* and other species. *J. Plant Res.* 116 241–252. 10.1007/s10265-003-0096-4 12721786

[B153] Shimizu-Sato S. Tanaka M. Mori H. (2008). Auxin–cytokinin interactions in the control of shoot branching. *Plant Mol. Biol.* 69 429–435. 10.1007/s11103-008-9416-3 18974937

[B154] Shuai B. Reynaga-Peña C. G. Springer P. S. (2002). The lateral organ boundaries gene defines a novel, plant-specific gene family. *Plant Physiol.* 129 747–761. 10.1104/pp.010926 12068116 PMC161698

[B155] Šimášková M. O’Brien J. A. Khan M. Van Noorden G. Ötvös K. Vieten A. (2015). Cytokinin response factors regulate *PIN-FORMED* auxin transporters. *Nat. Commun.* 6:8717. 10.1038/ncomms9717 26541513

[B156] Šmehilová M. Dobrùšková J. Novák O. Takáč T. Galuszka P. (2016). Cytokinin-specific glycosyltransferases possess different roles in cytokinin homeostasis maintenance. *Front. Plant Sci.* 7:1264. 10.3389/fpls.2016.01264 27602043 PMC4993776

[B157] Šmehilová M. Galuszka P. Bilyeu K. D. Jaworek P. Kowalska M. Šebela M. (2009). Subcellular localization and biochemical comparison of cytosolic and secreted cytokinin dehydrogenase enzymes from maize. *J. Exp. Bot.* 60 2701–2712. 10.1093/jxb/erp126 19436049

[B158] Smit M. E. McGregor S. R. Sun H. Gough C. Bågman A.-M. Soyars C. L. (2020). A PXY-mediated transcriptional network integrates signaling mechanisms to control vascular development in *Arabidopsis*. *Plant Cell* 32 319–335. 10.1105/tpc.19.00562 31806676 PMC7008486

[B159] Sottosanto J. B. Gelli A. Blumwald E. (2004). DNA array analyses of *Arabidopsis thaliana* lacking a vacuolar Na+/H+ antiporter: impact of AtNHX1 on gene expression. *Plant J.* 40 752–771. 10.1111/j.1365-313X.2004.02253.x 15546358

[B160] Sparkes A. De Baetselier P. Roelants K. De Trez C. Magez S. Van Ginderachter J. A. (2017). The non-mammalian MIF superfamily. *Immunobiology* 222 473–482. 10.1016/j.imbio.2016.10.006 27780588 PMC5293613

[B161] Striberny B. Melton A. E. Schwacke R. Krause K. Fischer K. Goertzen L. R. (2017). Cytokinin response factor 5 has transcriptional activity governed by its C-terminal domain. *Plant Signal. Behav.* 12:e1276684. 10.1080/15592324.2016.1276684 28045578 PMC5351726

[B162] Stührwohldt N. Dahlke R. I. Kutschmar A. Peng X. Sun M.-X. Sauter M. (2015). Phytosulfokine peptide signaling controls pollen tube growth and funicular pollen tube guidance in *Arabidopsis thaliana*. *Physiol. Plant.* 153 643–653. 10.1111/ppl.12270 25174442

[B163] Sun L. Feraru E. Feraru M. I. Waidmann S. Wang W. Passaia G. (2020). PIN-LIKES coordinate brassinosteroid signaling with nuclear auxin input in *Arabidopsis thaliana*. *Curr. Biol.* 30 1579.e6–1588.e6. 10.1016/j.cub.2020.02.002 32169207 PMC7198975

[B164] Sun X. Malhis N. Zhao B. Xue B. Gsponer J. Rikkerink E. H. A. (2020). Computational disorder analysis in ethylene response factors uncovers binding motifs critical to their diverse functions. *Int. J. Mol. Sci.* 21:74. 10.3390/ijms21010074 31861935 PMC6981732

[B165] Sun L. Zhang A. Zhou Z. Zhao Y. Yan A. Bao S. (2015). GLABROUS INFLORESCENCE STEMS3 (GIS3) regulates trichome initiation and development in *Arabidopsis*. *New Phytol.* 206 220–230. 10.1111/nph.13218 25640859

[B166] Takei K. Sakakibara H. Sugiyama T. (2001a). Identification of genes encoding adenylate isopentenyltransferase, a cytokinin biosynthesis enzyme, in *Arabidopsis thaliana*. *J. Biol. Chem.* 276 26405–26410. 10.1074/jbc.M102130200 11313355

[B167] Takei K. Sakakibara H. Taniguchi M. Sugiyama T. (2001b). Nitrogen-dependent accumulation of cytokinins in root and thetranslocation to leaf: implication of cytokinin species that induces geneexpression of maize responseregulator. *Plant Cell Physiol.* 42 85–93. 10.1093/pcp/pce009 11158447

[B168] Takei K. Ueda N. Aoki K. Kuromori T. Hirayama T. Shinozaki K. (2004a). AtIPT3 is a key determinant of nitrate-dependent cytokinin biosynthesis in *Arabidopsis*. *Plant Cell Physiol.* 45 1053–1062. 10.1093/pcp/pch119 15356331

[B169] Takei K. Yamaya T. Sakakibara H. (2004b). Arabidopsis CYP735A1 and CYP735A2 encode cytokinin hydroxylases that catalyze the biosynthesis of trans-zeatin. *J. Biol. Chem.* 279 41866–41872. 10.1074/jbc.M406337200 15280363

[B170] Taniguchi M. Sasaki N. Tsuge T. Aoyama T. Oka A. (2007). ARR1 directly activates cytokinin response genes that encode proteins with diverse regulatory functions. *Plant Cell Physiol.* 48 263–277. 10.1093/pcp/pcl063 17202182

[B171] Teakle G. R. Manfield I. W. Graham J. F. Gilmartin P. M. (2002). Arabidopsis thaliana GATA factors: organisation, expression and DNA-binding characteristics. *Plant Mol. Biol.* 50 43–56. 10.1023/A:1016062325584 12139008

[B172] Thilmony R. Underwood W. He S. Y. (2006). Genome-wide transcriptional analysis of the *Arabidopsis thaliana* interaction with the plant pathogen *Pseudomonas syringae* pv. tomato DC3000 and the human pathogen *Escherichia coli* O157:H7. *Plant J.* 46 34–53. 10.1111/j.1365-313X.2006.02725.x 16553894

[B173] To J. P. C. Haberer G. Ferreira F. J. Deruère J. Mason M. G. Schaller G. E. (2004). Type-A *Arabidopsis* response regulators are partially redundant negative regulators of cytokinin signaling. *Plant Cell* 16 658–671. 10.1105/tpc.018978 14973166 PMC385279

[B174] Tran L.-S. P. Urao T. Qin F. Maruyama K. Kakimoto T. Shinozaki K. (2007). Functional analysis of AHK1/ATHK1 and cytokinin receptor histidine kinases in response to abscisic acid, drought, and salt stress in *Arabidopsis*. *Proc. Natl. Acad. Sci. U.S.A.* 104 20623–20628. 10.1073/pnas.0706547105 18077346 PMC2154481

[B175] Urao T. Yakubov B. Satoh R. Yamaguchi-Shinozaki K. Seki M. Hirayama T. (1999). A transmembrane hybrid-type histidine kinase in *Arabidopsis* functions as an osmosensor. *Plant Cell* 11 1743–1754. 10.1105/tpc.11.9.1743 10488240 PMC144312

[B176] Vega A. O’Brien J. A. Gutiérrez R. A. (2019). Nitrate and hormonal signaling crosstalk for plant growth and development. *Curr. Opin. Plant Biol.* 52 155–163. 10.1016/j.pbi.2019.10.001 31726384

[B177] Wallmeroth N. Anastasia A. K. Harter K. Berendzen K. W. Mira-Rodado V. (2017). *Arabidopsis* response regulator 22 inhibits cytokinin-regulated gene transcription in vivo. *Protoplasma* 254 597–601. 10.1007/s00709-016-0944-4 26769709

[B178] Wallmeroth N. Jeschke D. Slane D. Nägele J. Veerabagu M. Mira-Rodado V. (2019). ARR22 overexpression can suppress plant two-component regulatory systems. *PLoS One* 14:e0212056. 10.1371/journal.pone.0212056 30742656 PMC6370222

[B179] Wang J. Ma X.-M. Kojima M. Sakakibara H. Hou B.-K. (2011). N-Glucosyltransferase UGT76C2 is involved in cytokinin homeostasis and cytokinin response in *Arabidopsis thaliana*. *Plant Cell Physiol.* 52 2200–2213. 10.1093/pcp/pcr152 22051886

[B180] Wang Y. Li L. Ye T. Zhao S. Liu Z. Feng Y.-Q. (2011). Cytokinin antagonizes ABA suppression to seed germination of *Arabidopsis* by downregulating ABI5 expression. *Plant J.* 68 249–261. 10.1111/j.1365-313X.2011.04683.x 21699589

[B181] Wang J. Ma X.-M. Kojima M. Sakakibara H. Hou B.-K. (2013). Glucosyltransferase UGT76C1 finely modulates cytokinin responses via cytokinin N-glucosylation in *Arabidopsis thaliana*. *Plant Physiol. Biochem.* 65 9–16. 10.1016/j.plaphy.2013.01.012 23416491

[B182] Wang R. Okamoto M. Xing X. Crawford N. M. (2003). Microarray analysis of the nitrate response in *Arabidopsis* roots and shoots reveals over 1,000 rapidly responding genes and new linkages to glucose, Trehalose-6-Phosphate, iron, and sulfate metabolism. *Plant Physiol.* 132 556–567. 10.1104/pp.103.021253 12805587 PMC166997

[B183] Wang R. Tischner R. Gutiérrez R. A. Hoffman M. Xing X. Chen M. (2004). Genomic analysis of the nitrate response using a nitrate reductase-null mutant of *Arabidopsis*. *Plant Physiol.* 136 2512–2522. 10.1104/pp.104.044610 15333754 PMC523318

[B184] Wang X. Lin S. Liu D. Gan L. McAvoy R. Ding J. (2020). Evolution and roles of cytokinin genes in angiosperms 1: do ancient IPTs play housekeeping while non-ancient IPTs play regulatory roles? *Horticult. Res.* 7:28. 10.1038/s41438-019-0211-x 32140237 PMC7049300

[B185] Weigel D. (1995). The APETALA2 domain is related to a novel type of DNA binding domain. *Plant Cell* 7 388–389. 10.1105/tpc.7.4.388 7773013 PMC160790

[B186] Werner T. Holst K. Pörs Y. Guivarc’h A. Mustroph A. Chriqui D. (2008). Cytokinin deficiency causes distinct changes of sink and source parameters in tobacco shoots and roots. *J. Exp. Bot.* 59 2659–2672. 10.1093/jxb/ern134 18515826 PMC2486470

[B187] Werner T. Köllmer I. Bartrina I. Holst K. Schmülling T. (2006). New insights into the biology of cytokinin degradation. *Plant Biol.* 8 371–381. 10.1055/s-2006-923928 16807830

[B188] Werner T. Motyka V. Laucou V. Smets R. Van Onckelen H. Schmülling T. (2003). Cytokinin-deficient transgenic arabidopsis plants show multiple developmental alterations indicating opposite functions of cytokinins in the regulation of shoot and root meristem activity. *Plant Cell* 15 2532–2550. 10.1105/tpc.014928 14555694 PMC280559

[B189] Werner T. Motyka V. Strnad M. Schmülling T. (2001). Regulation of plant growth by cytokinin. *Proc. Natl. Acad. Sci. U.S.A.* 98 10487–10492. 10.1073/pnas.171304098 11504909 PMC56987

[B190] Werner T. Schmülling T. (2009). Cytokinin action in plant development. *Curr. Opin. Plant Biol.* 12 527–538. 10.1016/j.pbi.2009.07.002 19740698

[B191] Wohlbach D. J. Quirino B. F. Sussman M. R. (2008). Analysis of the *Arabidopsis* histidine kinase ATHK1 reveals a connection between vegetative osmotic stress sensing and seed maturation. *Plant Cell* 20 1101–1117. 10.1105/tpc.107.055871 18441212 PMC2390728

[B192] Wybouw B. De Rybel B. (2019). Cytokinin – a developing story. *Trends Plant Sci.* 24 177–185. 10.1016/j.tplants.2018.10.012 30446307

[B193] Yamakawa S. Matsubayashi Y. Sakagami Y. Kamada H. Satoh S. (1998a). Promotion by a peptidyl growth factor, phytosulfokine, of chlorophyll formation in etiolated cotyledon of cucumber. *Biosci. Biotechnol. Biochem.* 62 2441–2443. 10.1271/bbb.62.2441 27392403

[B194] Yamakawa S. Sakuta C. Matsubayashi Y. Sakagami Y. Kamada H. Satoh S. (1998b). The promotive effects of a peptidyl plant growth factor, phytosulfokine-α, on the formation of adventitious roots and expression of a gene for a root-specific cystatin in cucumber hypocotyls. *J. Plant Res.* 111 453–458. 10.1007/BF02507810

[B195] Yamakawa S. Matsubayashi Y. Sakagami Y. Kamada H. Satoh S. (1999). Promotive effects of the peptidyl plant growth factor, phytosulfokine-α, on the growth and chlorophyll content of *Arabidopsis* seedlings under high night-time temperature conditions. *Biosci. Biotechnol. Biochem.* 63 2240–2243. 10.1271/bbb.63.2240 10664861

[B196] Yang B. Song Z. Li C. Jiang J. Zhou Y. Wang R. (2018). RSM1, an *Arabidopsis* MYB protein, interacts with HY5/HYH to modulate seed germination and seedling development in response to abscisic acid and salinity. *PLoS Genet.* 14:e1007839. 10.1371/journal.pgen.1007839 30566447 PMC6317822

[B197] Yang C. Li L. (2017). Hormonal regulation in shade avoidance. *Front. Plant Sci.* 8:1527. 10.3389/fpls.2017.01527 28928761 PMC5591575

[B198] Yang G. Shen S. Kobayashi T. Matsubayashi Y. Sakagami Y. Kamada H. (1999). Stimulatory effects of a novel peptidyl plant growth factor, phytosulfokine-α, on the adventitious bud formation from callus of *Antirrhinum majus*. *Plant Biotechnol.* 16 231–234. 10.5511/plantbiotechnology.16.231

[B199] Yang H. Matsubayashi Y. Nakamura K. Sakagami Y. (1999). *Oryza sativa PSK* gene encodes a precursor of phytosulfokine-α, a sulfated peptide growth factor found in plants. *Proc. Natl. Acad. Sci. U.S.A.* 96 13560–13565. 10.1073/pnas.96.23.13560 10557360 PMC23987

[B200] Yokoyama A. Yamashino T. Amano Y.-I. Tajima Y. Imamura A. Sakakibara H. (2007). Type-B ARR transcription factors, ARR10 and ARR12, are implicated in cytokinin-mediated regulation of protoxylem differentiation in roots of *Arabidopsis thaliana*. *Plant Cell Physiol.* 48 84–96. 10.1093/pcp/pcl040 17132632

[B201] Zhang H. Lin X. Han Z. Wang J. Qu L.-J. Chai J. (2016). SERK family receptor-like kinases function as co-receptors with PXY for plant vascular development. *Mol. Plant* 9 1406–1414. 10.1016/j.molp.2016.07.004 27449136

[B202] Zhang K. Wang R. Zi H. Li Y. Cao X. Li D. (2018). Auxin response factor3 regulates floral meristem determinacy by repressing cytokinin biosynthesis and signaling. *Plant Cell* 30 324–346. 10.1105/tpc.17.00705 29371438 PMC5868698

[B203] Zhou Z.-Y. Zhang C.-G. Wu L. Zhang C.-G. Chai J. Wang M. (2011). Functional characterization of the *CKRC1/TAA1* gene and dissection of hormonal actions in the *Arabidopsis* root. *Plant J.* 66 516–527. 10.1111/j.1365-313X.2011.04509.x 21255165

[B204] Zhou Z. An L. Sun L. Zhu S. Xi W. Broun P. (2011). Zinc finger protein5 is required for the control of trichome initiation by acting upstream of zinc finger protein8 in *Arabidopsis*. *Plant Physiol.* 157 673–682. 10.1104/pp.111.180281 21803862 PMC3192550

[B205] Zubo Y. O. Blakley I. C. Franco-Zorrilla J. M. Yamburenko M. V. Solano R. Kieber J. J. (2018). Coordination of chloroplast development through the action of the GNC and GLK transcription factor families. *Plant Physiol.* 178 130–147. 10.1104/pp.18.00414 30002259 PMC6130010

[B206] Zubo Y. O. Blakley I. C. Yamburenko M. V. Worthen J. M. Street I. H. Franco-Zorrilla J. M. (2017). Cytokinin induces genome-wide binding of the type-B response regulator ARR10 to regulate growth and development in *Arabidopsis*. *Proc. Natl. Acad. Sci. U.S.A.* 114 E5995–E6004. 10.1073/pnas.1620749114 28673986 PMC5530654

[B207] Zürcher E. Müller B. (2016). “Cytokinin synthesis, signaling, and function—advances and new insights,” in *International Review of Cell and Molecular Biology*, ed. Jeon K. W. (Cambridge, MA: Academic Press), 1–38. 10.1016/bs.ircmb.2016.01.001 27017005

[B208] Zwack P. J. De Clercq I. Howton T. C. Hallmark H. T. Hurny A. Keshishian E. A. (2016). Cytokinin response factor 6 represses cytokinin-associated genes during oxidative stress. *Plant Physiol.* 172 1249–1258. 10.1104/pp.16.00415 27550996 PMC5047073

[B209] Zwack P. J. Rashotte A. M. (2013). Cytokinin inhibition of leaf senescence. *Plant Signal. Behav.* 8:e24737. 10.4161/psb.24737 23656876 PMC3908980

